# Complete chloroplast genomes shed light on phylogenetic relationships, divergence time, and biogeography of Allioideae (Amaryllidaceae)

**DOI:** 10.1038/s41598-021-82692-5

**Published:** 2021-02-05

**Authors:** Ju Namgung, Hoang Dang Khoa Do, Changkyun Kim, Hyeok Jae Choi, Joo‑Hwan Kim

**Affiliations:** 1 Department of Life Science, Gachon University, Seongnam, 13120 Republic of Korea; 2 Nguyen Tat Thanh Hi-Tech Institute, Nguyen Tat Thanh University, Ho Chi Minh City, Vietnam; 3 Department of Biology and Chemistry, Changwon National University, Gyeongsangnamdo, 51140 Republic of Korea

**Keywords:** Phylogenetics, Molecular evolution, Plant sciences

## Abstract

Allioideae includes economically important bulb crops such as garlic, onion, leeks, and some ornamental plants in Amaryllidaceae. Here, we reported the complete chloroplast genome (cpDNA) sequences of 17 species of Allioideae, five of Amaryllidoideae, and one of Agapanthoideae. These cpDNA sequences represent 80 protein-coding, 30 tRNA, and four rRNA genes, and range from 151,808 to 159,998 bp in length. Loss and pseudogenization of multiple genes (i.e., *rps2, infA*, and *rpl22*) appear to have occurred multiple times during the evolution of Alloideae. Additionally, eight mutation hotspots, including *rps15-ycf1*, *rps16-trnQ-UUG*, *petG-trnW-CCA*, *psbA* upstream, *rpl32-trnL-UAG*, *ycf1*, *rpl22*, *matK*, and *ndhF*, were identified in the studied *Allium* species. Additionally, we present the first phylogenomic analysis among the four tribes of Allioideae based on 74 cpDNA coding regions of 21 species of Allioideae, five species of Amaryllidoideae, one species of Agapanthoideae, and five species representing selected members of Asparagales. Our molecular phylogenomic results strongly support the monophyly of Allioideae, which is sister to Amaryllioideae. Within Allioideae, Tulbaghieae was sister to Gilliesieae-Leucocoryneae whereas Allieae was sister to the clade of Tulbaghieae- Gilliesieae-Leucocoryneae. Molecular dating analyses revealed the crown age of Allioideae in the Eocene (40.1 mya) followed by differentiation of Allieae in the early Miocene (21.3 mya). The split of Gilliesieae from Leucocoryneae was estimated at 16.5 mya. Biogeographic reconstruction suggests an African origin for Allioideae and subsequent spread to Eurasia during the middle Eocene. Cool and arid conditions during the late Eocene led to isolation between African and Eurasian species. African Allioideae may have diverged to South American taxa in the late Oligocene. Rather than vicariance, long-distance dispersal is the most likely explanation for intercontinental distribution of African and South American Allioideae species.

## Introduction

Allioideae Herbert, a subfamily of Amaryllidaceae (Asparagales), comprises four tribes, 13 genera and over 900 species^1^. The subfamily is widely distributed in temperate and subtropical regions of the Northern Hemisphere and South America, and occurs locally in South Africa^2^. Most Allioideae are economically important plants used in traditional medicine, horticulture, and also as ornamentals. Within Amaryllidaceae, Allioideae can easily be distinguished from the other subfamilies based on superior ovary and solid styles. These subfamilies are further characterized by possession of unique chemical compounds^3^. Molecular phylogenetic studies have demonstrated the monophyly of each subfamily of Amaryllidaceae using chloroplast (cp) DNA sequence data^4–6^. Despite the morphological, anatomical, chemical, and molecular distinctiveness of Allioideae, its sister group is controversial. Meerow et al.^4^ suggested that Allioideae is sister to the Agapanthoideae –Amaryllidoideae clade based on two cpDNA *rbcL* and *trnL-F* regions, which was also reported by Costa et al.^7^ inferred from four loci dataset. A more recent analysis of four cpDNA genes by Chen et al.^6^ found support for a sister relationship between Allioideae and Amaryllidoideae, which is in agreement with the results of Steele et al.^5^ and Xie et al.^8^. Although these studies shed light on the molecular systematics of Amaryllidaceae, comprehensive phylogenetic analysis using complete cp genome sequences has not yet been conducted to resolve the systematic position of Allioideae within the family.

Within Allioideae, four tribes (Allieae, Gilliesieae, Leucocoryneae, and Tulbaghieae) are recognized based on the presence or absence of corona, flower symmetry, style position, and the presence or absence of septal nectaries^1,9^. For example, Allieae, comprising a single genus (*Allium*) classified in 15 subgenera^9^, is defined by a combination of traits including a gynobasic style, actinomorphic flowers, and absence of corona and sepal nectaries. Previous molecular phylogenetic analyses of Allioideae have shown that each tribe forms a well-supported clade^10,11^. However, disagreement about the relationships among tribes Gilliesieae, Tulbaghieae, and Leucocoryneae within Allioideae continues. Souza et al.^11^ examined phylogenetic relationships within Allioideae using combined nuclear ribosomal internal transcribed spacer (nrITS) and single cpDNA marker (*trnG* intron) sequences, and revealed that Gilliesieae is closely related to the Tulbaghieae–Leucocoryneae clade. Later, Sassone and Giussani^2^ revealed that Tulbaghieae is sister to the Gilliesieae-Leucocoryneae clade based on nrITS and two cpDNA (*ndhF* and *matK*) sequences, which is in consonance with the results of Costa et al.^7^. These studies focused on the tribe and genus levels and used limited DNA regions. Thus, the phylogenetic relationships among tribes within Allioideae could be clarified by including more DNA regions.

Understanding the disjunct distribution pattern of plant groups has long been a major focus of biogeography^7,12^. Within the molecular phylogenetic framework, biogeographic origin and migration routes leading to the present disjunct distributions of a variety of plant taxa have been inferred^13^. In particular, study of the biogeographic history of plants with disjunct distributions between the Northern and Southern Hemispheres is informative, as it can provide information about global biodiversity. Two main migration routes between the Northern and Southern Hemispheres have been recognized: one between North and South America and the others between Europe and Africa several times in the Tertiary^14,15^. Migration between Asia and Australia in the Miocene and later has been reported, but less commonly^16^. In Allioideae, Allieae is widely distributed in the Northern Hemisphere including Eurasia and North America, while all other tribes are endemic to South Africa or South America^2,10^. Tulbaghieae is endemic to South Africa; Gilliesieae and Leucocoryneae are restricted to South America, with one exception, *Nothoscordum bivalve* (L.) Britton, which has expanded its range as far as the southern half of the USA. Therefore, the distribution pattern of Allioideae offers an ideal opportunity for understanding the biogeographic origins and migration routes of plant groups showing disjunct distribution between the two hemispheres. To determine the migration patterns of these Northern–Southern Hemisphere disjunct species, the most efficient way is to estimate their divergence times using DNA sequences and resolved phylogenies^15^. Several studies have estimated the divergence time of the major clades of Allioideae using DNA sequences^2,6–8,17^. Chen et al.^6^ used four cpDNA coding regions to estimate the divergence times of families and major subfamilies of Asparagales. They suggested that the crown node of Allioideae occurred 37 million years ago (mya) in the late Eocene. Li et al.^17^ and Sassone and Giussani^2^ estimated the crown nodes of Allioideae tribes between 18 (Gilliesieae) and 34 mya (Allieae). However, these studies did not determine the divergence times of taxa that are distributed disjunctly between the Northern and Southern Hemispheres. The latest study on evolutionary history of Allioideae revealed an ‘out of India’ origin of Allieae before a widespread distribution in the northern hemisphere based on the nrITS and three cpDNA (*matK, ndhF*, and *rbcL*) sequence data^7^.

The chloroplast genome (cpDNA), being inherited maternally (> 85%), paternally, or biparentally and containing coding genes necessary for photosynthesis, provides useful data for phylogenetic studies, biogeographical analyses, and reconstruction of the evolutionary history of angiosperms^18–20^. Numerous studies have been conducted on the complete cpDNA of Allioideae. However, these investigations focused on few species or small groups within Allioideae^8,21,22^. Therefore, the uncertainties of Allioideae phylogeny have not been fully resolved with regard to subgeneric, tribal, and subfamilial relationships. Here, we sequenced 23 chloroplast genomes representing four tribes of Allioideae using next-generation sequencing (NGS) technology. Using these results together with published cpDNA data for Asparagaceae, Xanthorrhoeaceae, and Iridaceae, we aim to (1) explore cpDNA evolution in Allioideae; (2) clarify the tribal and subfamilial relationships of Allioideae and related taxa; (3) estimate the divergence times of Allioideae; and (4) reconstruct the biogeographic history of the subfamily.

## Results

### Comparative analysis of cpDNA features in Allioideae and related taxa

**Figure 1:**
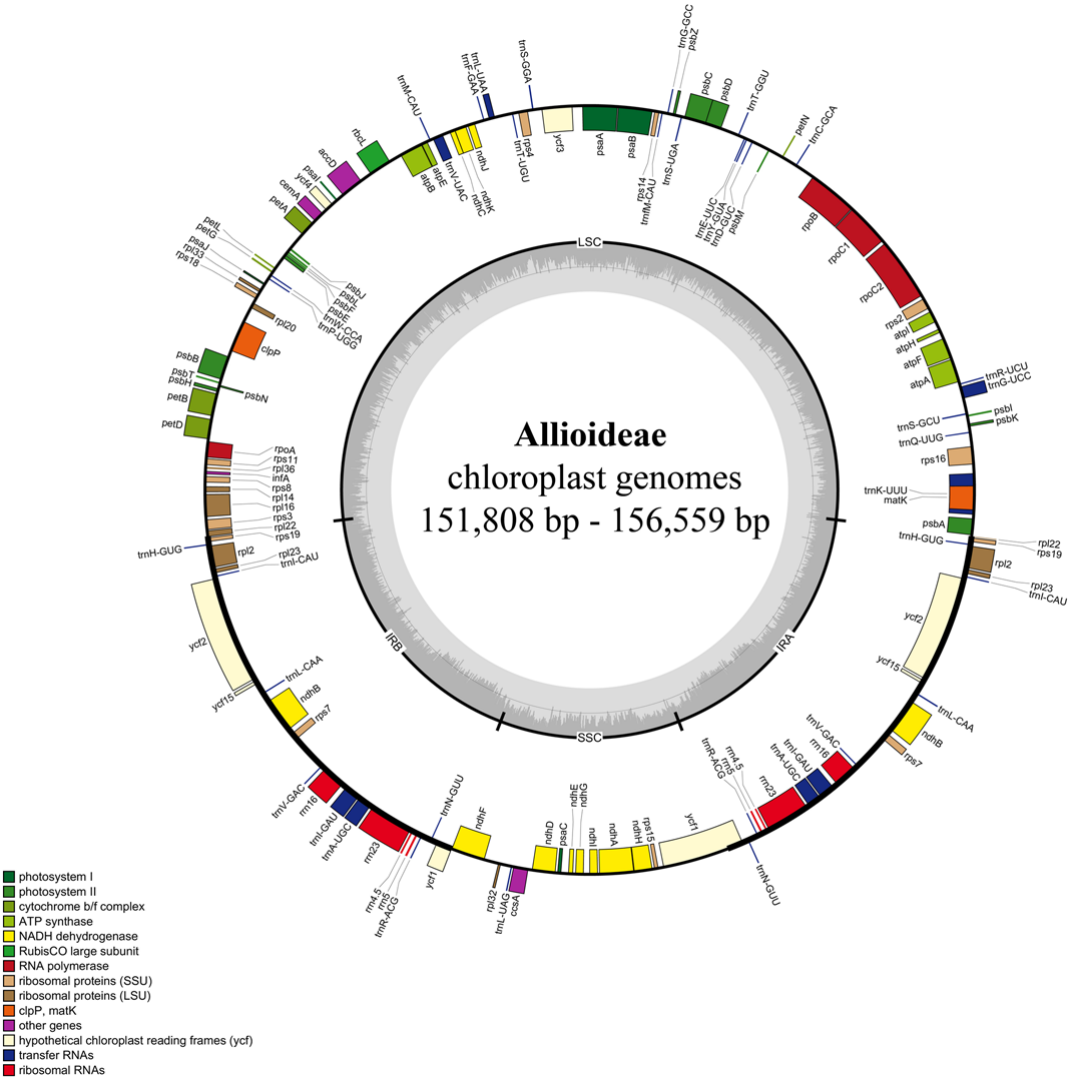
Representative map of the plastid genome of Allioideae species. Genes inside the circle are transcribed clockwise, whereas those outside the circle are transcribed counter-clockwise. *LSC* large single copy, *SSC* small single copy, *IRA-IRB* inverted repeat regions.

The cpDNA genomes of Allioideae have a quadripartite structure that includes a large single copy (LSC), a small single copy (SSC), and two inverted repeat (IR) regions (Fig. 1). However, cpDNA genome size varies from 145,819 to 157,735 bp (Table 1). Among the four tribes of Allioideae, Allieae species have the smallest cpDNA (*Allium paradoxum;* 145,819 bp) and the largest cpDNA (*Allium tuberosum;* 157,735 bp). Most cpDNA of Allioideae is smaller than those of Agapanthoideae (157,055 bp) and Amaryllidoideae (ranging from 158,355 to 159,998 bp). Additionally, the GC content of cpDNA sequence in *Allium* species (generally ≤ 37.1%) is lower than those of other examined taxa (Table 1).

**Table 1:** Some features of chloroplast genomes in Allioideae and related species.

Taxa	Accession number	Total length		Number of genes/ pseudogenes	tRNA	rRNA	LSC-IR junction	SSC-IR junction
		Length	%GC					
*Allium siculum*	MT348457	154,225	36.9	80/3	30	4	*rpl22* (35 bp)	*ycf1* (1066 bp) gap (151 bp)
*A. monanthum*	MT348452	154,306	36.9	78ab/2	30	4	IGS (*rps19-rpl22*) 19 bp	*ycf1* (1136 bp) gap (26 bp)
*A. ursinum*	MH157875	153,252	37	80/2	30	4	*rpl22* (20 bp)	*ycf1-ndhF* overlap (55 bp)
*A. cernuum*	MT348440	153,750	36.9	80/1	30	4	*rpl22* (39 bp)	*ycf1-ndhF* overlap (48 bp)
*A. neriniflorum*	MT348443	154,129	36.9	80/2	30	4	*rpl22* (34 bp)	*ycf1* (1092 bp) gap (176 bp)
*A. victorialis*	MF687749	154,074	37	80/1	30	4	*rpl22* (36 bp)	*ycf1-ndhF* overlap (31 bp)
*A. tricoccum*	MT348456	153,565	37.1	80/1	30	4	*rpl22* (34 bp)	*ycf1-ndhF* overlap (31 bp)
*A. ochotense*	MT348451	154,048	37	80/1	30	4	*rpl22* (34 bp)	*ycf1-ndhF* overlap (31 bp)
*A. karataviense*	MT348442	151,808	37	79a/2	30	4	*rps19* (10 bp)	*ycf1* (1237 bp) gap (31 bp)
*A.nigrum*	MT348458	152,241	36.7	80/3	30	4	*rpl22* (32 bp)	*ycf1* (1078 bp) gap (60 bp)
*A. cyathophorum*	MT348441	153,503	36.8	80/1	30	4	*rpl22* (39 bp)	*ycf1* (1054 bp) gap (18 bp)
*A. spicatum*	MT348453	152,822	36.9	79a/2	30	4	*rps19* (10 bp)	*ycf1* (1221 bp) gap (15 bp)
*A. senescens*	MT348450	153,542	36.8	80/1	30	4	*rpl22* (21 bp)	*ycf1* (1070 bp) gap (30 bp)
*A. sativum*	NC031829	153,172	36.7	80/2	30	4	*rpl22* (34 bp)	*ycf1* (1070 bp) gap (52 bp)
*A. koreanum*	MT348449	153,162	36.8	80/2	30	4	*rpl22* (28 bp)	*ycf1-ndhF* overlap (9 bp)
*A. obliquum*	NC037199	152,387	36.8	80/2	30	4	*rpl22* (33 bp)	*ycf1* (1065 bp) gap (4 bp)
*A. cepa*	KM088013	153,529	36.8	80/2	30	4	*rpl22* (34 bp)	*ycf1-ndhF* overlap (1 bp)
*A. schoenoprasum*	MT348444	152,852	36.8	80/2	30	4	*rpl22* (34 bp)	*ycf1-ndhF* overlap (8 bp)
*A. altaicum*	MH159130	153,129	36.8	80/1	30	4	*rpl22* (33 bp)	*ycf1-ndhF* overlap (3 bp)
*A. ampeloprasum*	NC_044666	152,732	36.7	79a	30	4	*rpl22* (33 bp)	*ycf1* (1070 bp) gap (38 bp)
*A. fistulosum*	LT674586	152,859	36.9	80	30	4	*rpl22* (28 bp)	*ycf1* (1074 bp) gap (6 bp)
*A. macleanii*	LT699703	152,633	36.9	79a	30	4	*rpl22* (34 bp)	*ycf1* (1234 bp) gap (41 bp)
*A. paradoxum*	MH053150	145,819	37.1	77bcd/9	30	4	*rps3* (39 bp)	*ycf1* (522 bp)
*A. platyspathum*	LT673892	152,529	36.8	79e	30	4	*rps3* (45 bp)	*ycf1* (1066 bp) gap (13 bp)
*A. caeruleum*	MK820610	153,267	36.8	80/1	30	4	*rpl22* (16 bp)	*ycf1* (1038 bp) gap (29 bp)
*A. chrysocephalum*	MH992109	153,710	36.8	80/1	30	4	*rpl22* (16 bp)	*ycf1* (1041 bp) gap (2 bp)
*A. chrysanthum*	MH992108	153,621	36.8	80/1	30	4	*rpl22* (16 bp)	*ycf1* (1041 bp) gap (2 bp)
*A. chinense*	MK096442	152,525	36.8	80/1	30	4	*rpl22* (16 bp)	*ycf1* (1075 bp) gap (45 bp)
*A. fetisowii*	MK820612	154,018	36.9	80/1	30	4	*rpl22* (34 bp)	*ycf1* (1037 bp) gap (6 bp)
*A. forrestii*	MK820613	153,186	36.8	80/1	30	4	*rpl22* (15 bp)	*ycf1* (1043 bp) gap (12 bp)
*A. herderianum*	MH992110	153,605	36.8	80/1	30	4	*rpl22* (16 bp)	*ycf1* (1042 bp) gap (1 bp)
*A. macranthum*	MK820614	152,876	37.1	80/1	30	4	*rpl22* (34 bp)	*ycf1* (1031 bp) gap (33 bp)
*A. mairei*	MK820615	152,913	36.9	80/1	30	4	*rpl22* (34 bp)	*ycf1* (942 bp) gap (98 bp)
*A. maowenense*	MH992111	153,608	36.8	80/1	30	4	*rpl22* (16 bp)	*ycf1* (1042 bp) gap (1 bp)
*A. nanodes*	MK820616	154,077	37.0	80/4	30	4	*rpl22* (34 bp)	*ycf1* (1023 bp) gap (11 bp)
*A. oschaninii*	MK411816	153,580	36.8	80/1	30	4	*rpl22* (33 bp)	*ycf1* (1066 bp) gap (1 bp)
*A. polyrhizum*	MK820618	153,086	36.9	80/1	30	4	*rpl22* (16 bp)	*ycf1* (910 bp) gap (28 bp)
*A. prattii*	MG739457	154,482	37.0	80/3	30	4	*rpl22* (16 bp)	*ycf1* (1072 bp) gap (95 bp)
*A. przewalskianum*	MK820619	153,245	36.9	80/1	30	4	*rpl22* (16 bp)	*ycf1* (1021 bp) gap (10 bp)
*A. rude*	MH992112	153,697	36.7	80/1	30	4	*rpl22* (16 bp)	*ycf1* (1041 bp) gap (2 bp)
*A. schoenoprasides*	MK820620	153,583	36.7	80/1	30	4	*rpl22* (34 bp)	*ycf1* (1042 bp) gap (22 bp)
*A. praemixtum*	MK411817	153,226	36.8	80/1	30	4	*rpl22* (33 bp)	*ycf1* (1066 bp) gap (26 bp)
*A. pskemense*	MK411815	153,788	36.7	80	30	4	*rpl22* (33 bp)	*ycf1-ndhF* overlap (2 bp)
*A. strictum*	MK820622	152,962	36.8	80/1	30	4	*rpl22* (29 bp)	*ycf1* (1010 bp) gap (39 bp)
*A. tuberosum*	MK820623	157,735	36.9	80/1	30	4	*rpl22* (34 bp)	*ycf1* (1044 bp) gap (2 bp)
*A. xichuanense*	MH992113	153,673	36.7	80/1	30	4	*rpl22* (16 bp)	*ycf1* (1041 bp) gap (2 bp)
*Nothoscordum bonariense*	MT348455	156,559	37.5	80	30	4	IGS (*trnH-GUG-rps19*) 51 bp	*ycf1-ndhF* overlap (14 bp)
*Tulbagia violacea*	MT323239	156,449	37.6	80/3	30	4	*rpl22* (9 bp)	*ycf1-ndhF* overlap (50 bp)
*Gillesia graminea*	MT348447	154,067	37.8	80/1	30	4	*rpl22* (23 bp)	*ycf1-ndhF* overlap (29 bp)
*Agapanthus coddii*	MT348439	157,055	37.5	80	30	4	IGS (*rps19-rpl22*) 52 bp	*ycf1-ndhF* overlap (49 bp)
*Lycoris radiata*	MT348454	158,355	37.8	80	30	4	IGS (*rps19-rpl22*) 16 bp	*ycf1* (982 bp) gap (0 bp)
*Zephyranthes mesochloa*	MT323238	158,768	38	80	30	4	*rpl22* (3 bp)	*ycf1-ndhF* overlap (77 bp)
*Calostemma purpureum*	MT348445	159,500	37.7	80	30	4	*rpl22* (9 bp)	*ycf1-ndhF* overlap (38 bp)
*Crinum asiaticum* var *pendunculatum*	MT348448	158,683	37.8	80	30	4	*rpl22* (6 bp)	*ycf1-ndhF* overlap (8 bp)
*Cyrtanthus mackenii*	MT348446	159,998	37.6	80	30	4	*rpl22* (24 bp)	*ycf1-ndhF* overlap (3 bp)
*Asparagus officinalis*	NC034777	156,699	37.6	80	30	4	IGS (*rps19-rpl22*) 53 bp	*ycf1* (802 bp) gap (9 bp)
*Hosta yingeri*	NC039976	156,756	37.8	80/2	30	4	*rpl22* (29 bp)	*ycf1-ndhF* overlap (26 bp)
*Yucca filamentosa*	KX931467	157,785	37.8	80	30	4	IGS (*rps19-rpl22*) 26 bp	*ycf1* (959 bp) gap (9 bp)
*Xanthorrhorea preissii*	NC035996	158,116	37.9	80	30	4	IGS (*rps19-rpl22*) 46 bp	*ycf1-ndhF* overlap (51 bp)
*Iris sanguinea*	KT626943	150,862	38	80	30	4	IGS (*rps19-rpl22*) 37 bp	*ycf1-ndhF* overlap (49 bp)

a: *infA* loss; b: *rpl22* loss; c: *ndhF* loss; d: *ndhG* loss; e: *rps16* loss.

Although cpDNA size is variable, its gene content and order are quite stable among Allioideae and related taxa; it includes 80 protein-coding genes, 30 tRNAs, and four rRNAs (Fig. 1, Table 1, and Table S1). The loss of *infA* was observed in *Allium monanthum*, *A*. *karataviense*, *A*. *ampeloprasum, A*. *macleanii,* and *A*. *spicatum,* whereas complete deletion of *rpl22* and *rps16* was recorded in *A*. *monanthum* and *A. platyspathum*, respectively (Table 1). In addition to the loss of protein-coding regions, pseudogenization was annotated in some regions of the examined species, including *rps2* in most examined species of Allieae (excluding *A*. *fistulosum*, *A*. *macleanii*, *A*. *caeruleum*, *A*. *chinense*, *A*. *prattii*, and *A*. *pskemense*), *matK* (*A*. *karataviense*, *A*. *spicatum*, and *A*. *siculum*), *infA* (*A*. *monanthum*, *A*. *ochotense*, *A*. *tricoccum*, *A*. *siculum*, *A*. *victorialis*, *A*. *prattii, A*. *nanodes* and *Tulbaghia violacea*), *rps16* (*A*. *neriniflorum*, *A*. *schoenoprasum*, *A*. *ampeloprasum, A*. *chinense*, and *A*. *obliquum*), *rpl23* (*A*. *spicatum* and *T*. *violacea*), *accD* (*A*. *nigrum*, *A*. *cepa*, and *T*. *violacea*), *cemA* (*T*. *violacea*), *ycf2* (*A*. *neriniflorum*), *rpl36* (*A. caeruleum*), *atpB* and *rbcL* (*A. prattii* and *A. nanodes*) and *ycf1* (*Gilliesia graminea*). Notably, *A. paradoxum* possessed complete deletion of *rpl22, ndhF, ndhG*, and *rps2*, and pseudozenization of *infA, ndhJ, ndhK, ndhC, ndhD, ndhE, ndhI, ndhH,* and *ndhA* (Table 1). Also, pseudogenization of *ycf15* was observed in all examined chloroplast genomes of Amaryllidaceae and outgroups.

The boundaries between the LSC and IR regions are quite similar among Allioideae and other examined species, located in the coding region of *rpl22* (Table 1). However, the expansion lengths are variable, ranging from 3 to 39 bp. By contrast, the LSC-IR junction is within *rps19* (10 bp) in *A*. *karataviense* and *A*. *spicatum*. In *A*. *monanthum*, the LSC-IR border is in the intergenic spacer (IGS) between *rps19* and *rpl22*, which was also observed in *Agapanthus coddii*, *Lycoris radiata*, *Asparagus officinalis*, *Yucca filamentosa*, *Xanthorrhoea preissii*, and *Iris koreana*. Notably, *Nothoscordum bonariense* (Leucocoryneae) has a unique LSC-IR junction within the IGS between *trnH-GUG* and *rps19*. Similar to the variation of the LSC-IR border, three types of junction between SSC and IR regions were observed, including overlap, adjunction, and gap between *ycf1* and *ndhF*. Notably, adjunction was only found in *Lycoris radiata*. By contrast, the overlap and gap boundaries are common in Allioideae and related taxa (Table 1).

The Pi values of nucleotide diversity range from 0 to 0.08718 in *Allium* species and reach 0.09956 in Allioideae (Table S2) with the variation of noncoding regions being greater than that of coding sequences. In Allioideae, hotspot regions include *rps15-ycf1*, *rps16-trnQ-UUG*, *petG-trnW-CCA*, *psbA* upstream, *rpl32-trnL-UAG*, *ycf1*, *rpl22*, *matK*, and *ndhF*. Similarly, high variation in the DNA sequences of examined *Allium* species was found in *rps15-ycf1*, *petD-rpoA*, *petG-trnW-CCA*, *psbA* upstream, *rpl32-trnL-UAG*, *ycf1*, *infA*, *rps2*, and *ndhF*.

Analysis of repeats revealed 21 repeated regions in the cpDNA of Allioideae (Table S3). Most repeats are forward, aside from a palindromic repeat found only in *Allium koreanum* and *A*. *obliquum*. Additionally, repeats were abundant in noncoding regions. Some repeats were found in the *ycf2* and tRNA coding sequences (i.e., *trnF-GAA, trnA-UGC, trnfM-CAU-trnP-UGG, trnS-GCU,* and *trnS-UGA*). In addition to the shared repeats among Allioideae, unique repeats were found in *A*. *koreanum*, *A*. *cepa*, *A*. *obliquum*, *A*. *cyathophorum*, *A*. *nigrum*, *A*. *senescens*, and *A*. *ursinum* (Table S3).

A total of 72 regions containing simple sequence repeats (SSRs) were detected in the cpDNA of Allioideae, with lengths ranging from 10 to 20 bp (Table S4). Most SSRs are mononucleotide repeats made up of A and T nucleotides. These SSRs are located mostly in noncoding regions, except for repeats found in *ycf1*, *ycf2*, *rpoC1*, *rpoC2*, *ndhF*, *rps16*, and *cemA*. The number and lengths of SSRs varied among Allioideae species (Table S4).

### Phylogenetic relationships of Allioideae

**Figure 2:**
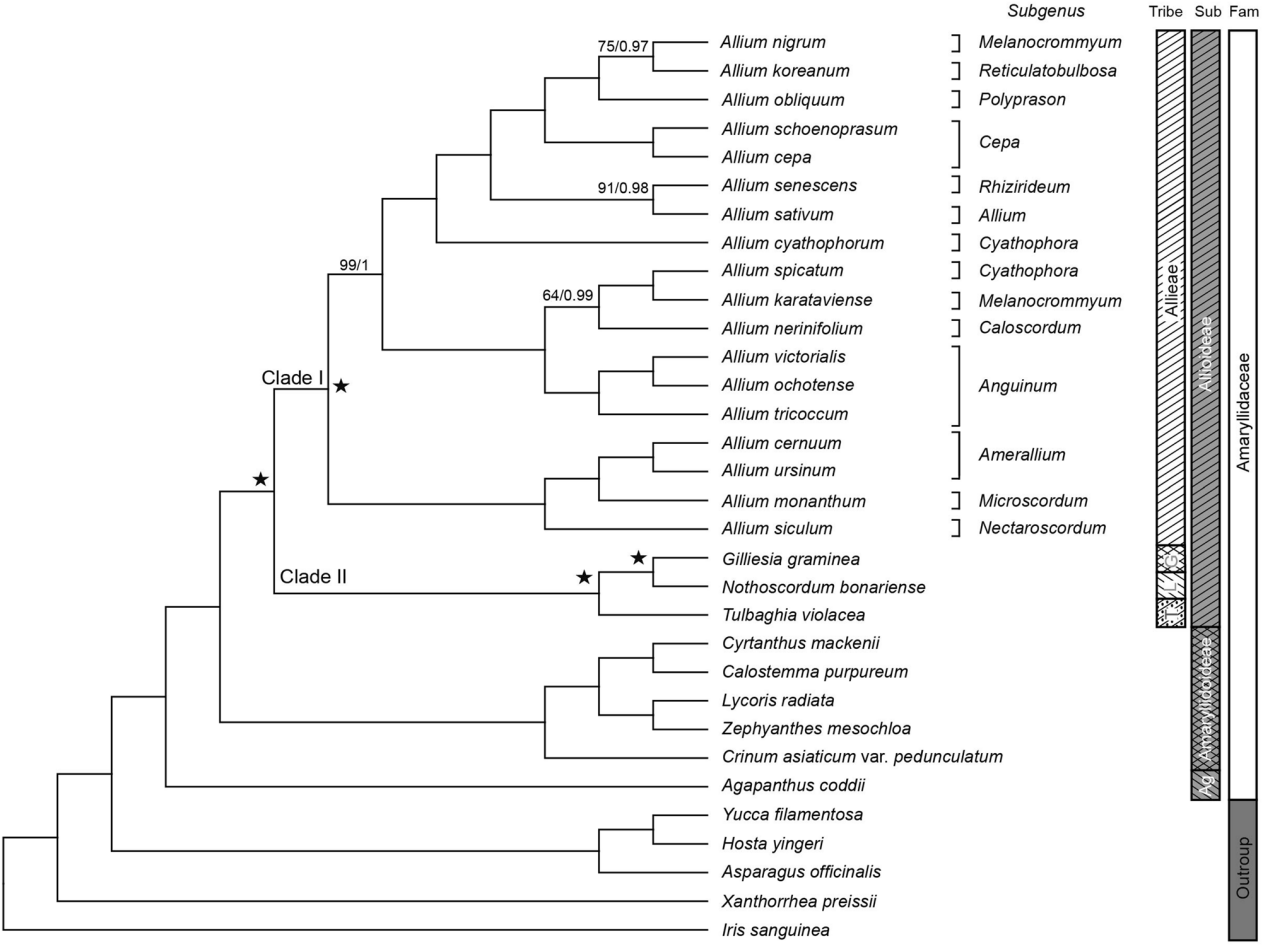
Maximum parsimony tree inferred from 74 protein-coding regions of 32 chloroplast genomes from Allioideae and related taxa. Numbers represent support values (bootstrap [BP]/ posterior probability [PP]). Only support values below BP = 100/PP = 1 are shown. Tri: Tribe; Sub: Subfamily; Fam: Family; T: Tulbaghieae; L: Leucocoryneae; G: Gilliesieae; Ag: Agapanthoideae. Clade I, Clade II, and black stars indicate interested points for further analyses.

Maximum Parsimony (MP) and Bayesian Inference (BI) analyses using 74 protein-coding regions of cpDNA produced trees with identical topology. The strict consensus tree gained from the MP analysis is shown in Fig. 2 (Tree length = 20,479; Consistency index (CI) = 0,7; Retention index (RI) = 0.8; Homoplasy index (HI) = 0.3). The monophyly of Allioideae was strongly supported. Amaryllidoideae was found to be sister to Allioideae with the highest support. Within Allioideae, Allieae (clade I) was sister to the clade II consisting of the remaining tribes. Within clade II, Tulbaghieae was sister to Gilliesieae-Leucocoryneae.

In *Allium*, three subclades (a-c) were recognized: “a” included *A. nigrum* through *A. cyathophorum*; “b” included *A. spicatum* through *A. tricoccum*; and “c” comprised *A. cernuum* through *A. siculum*. Although monophyly of three subgenera: *Cepa, Anguinum,* and *Anguinum* was supported, our results clearly demonstrate that two subgenera, *Cyathophora* and *Melanocrommyum*, represented by multiple species, were not monophyletic (Fig. 2).

### Molecular dating analyses

**Figure 3:**
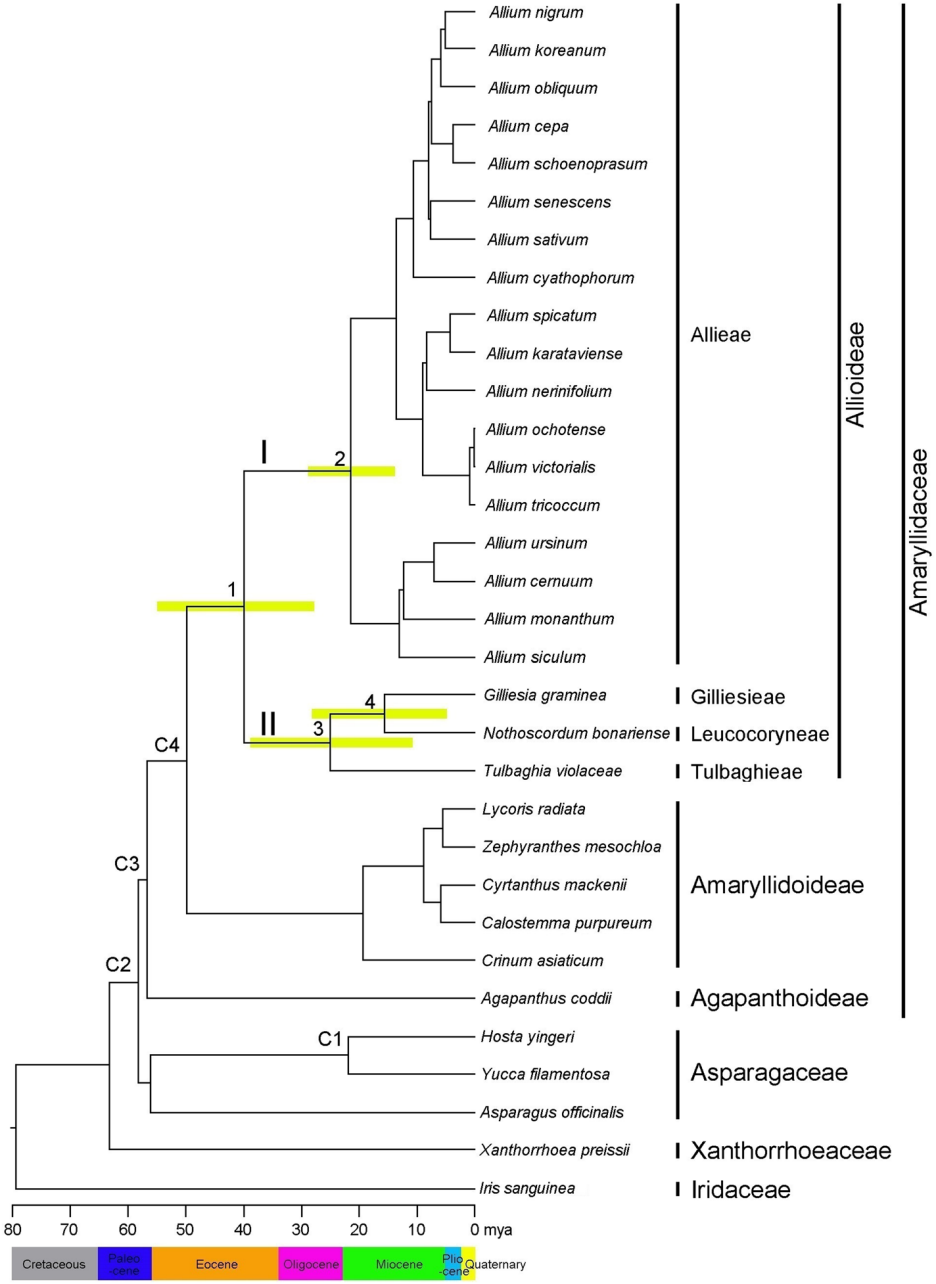
Chronogram showing divergence times estimated using BEAST based on data from 74 cpDNA sequences. Divergence times are shown for each node. Yellow bars represent 95% highest posterior density values for the estimated mean dates. The clades (I and II) correspond to those shown in Fig. 4. Nodes labelled C1–C4 are calibration points used for analysis (for details, see “Materials and Methods”). Numbers 1–4 indicate nodes of interest (for details, see Table 2).

Divergence time estimates for the tribes of Allioideae based on the combination of 74 coding gene sequences in the chloroplast genome are shown in Fig. 3 and Table 2. Our BEAST dating analysis resulted in estimates for the crown node of Allioideae (clade I) of 40.1 mya (95% highest posterior density [HPD] = 28.5–55.3 mya; node 1) in the Eocene. Within Allioideae, the age estimate for the crown node of Allieae in the Northern Hemisphere was 21.3 mya (95% HPD = 14.4–28.8 mya; node 2) in the early Miocene. The age estimate for the crown node of clade II, including the other tribes of Allioideae, was dated to 25.3 mya (95% HPD = 11.5–39.1 mya; node 3) in the late Oligocene. The divergence time between the Gilliesieae and Leucocoryneae was estimated at 16.5 mya (95% HPD = 5.0–28.5 mya; node 4) at the interface of the early and middle Miocene.

**Table 2:** Posterior age distributions of major nodes of Allioideae using BEAST, with results of ancestral area reconstruction using BBM and S-DIVA analyses.

Node^a^	Description	Age estimate (mya)		Ancestral area reconstruction (%)^b^	
		Mean	95% HPD	BBM	S-DIVA
1	Alloideae	40.1	28.5–55.3	C (82)	AC (50), ACD (50)
2	Allieae (clade I)	21.3	14.4–28.8	A (96)	A (100)
3	Gilliesieae + Leucocoryneae + Tulbaghieae (clade II)	25.3	11.5–39.1	C (89)	CD (100)
4	Gilliesieae + Leucocoryneae	16.5	5.0–28.5	D (91)	D (100)

Ancestral areas include: (A) Eurasia, (B) North America, (C) Africa, (D) South America, and (E) Australia.

**Figure 4:**
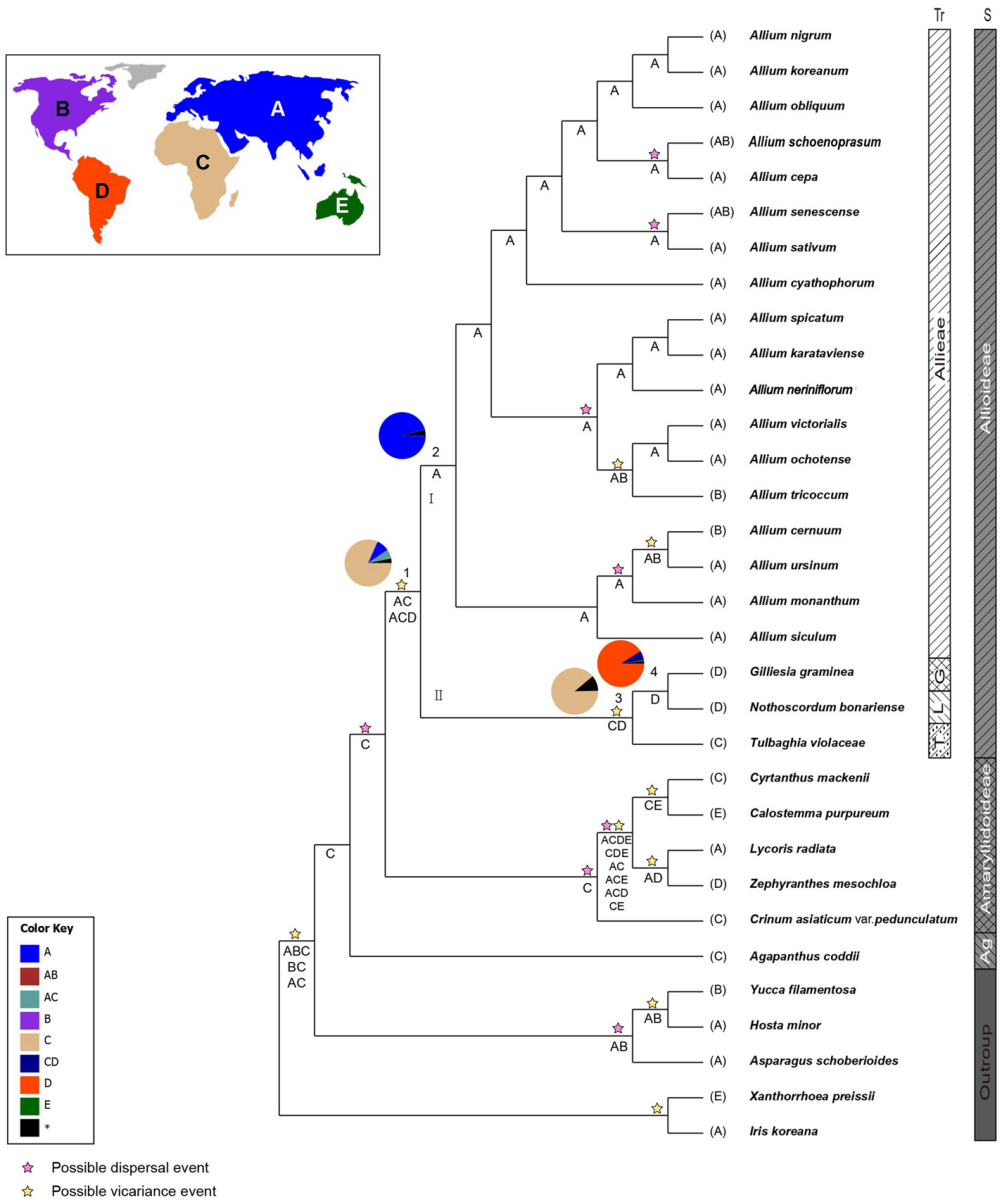
Summary of the Bayesian binary method (BBM) and statistical dispersal—vicariance analysis (S-DIVA) models of ancestral area reconstruction in Allioideae based on the BEAST combined-gene chronogram. The BBM ancestral area reconstructions with the highest likelihood are shown as pies for each Allioideae clade. Colour key for ancestral reconstruction at nodes of interest obtained from BBM analysis is provided in the figure. The results of S-DIVA reconstruction are indicated above the branches. The clades (I, II) and numbers (1–4) correspond to those in Fig. 3 (see Table 2 for details). Biogeographic regions used for BBM and S-DIVA analyses: A, Eurasia; B, North America; C, Africa; D, South America; and E, Australia.

^a^Node numbers and biogeographic codes correspond to those in Figs. 3, 4.

^b^Ancestral areas for each node are represented with marginal probability ≥ 10%.

### Ancestral area reconstruction

The ancestral ranges of the nodes of clades in Allioideae inferred using the Bayesian binary method (BBM) and statistical dispersal variance analysis (S-DIVA) are summarised in Fig. 4 and Table 2. The BBM reconstruction suggests that Africa (C) is the most probable ancestral area of Allioideae (node 1, 82%), whereas the S-DIVA range reconstruction for this node is Eurasia + Africa (AC, 50%) or Eurasia + Africa + South America (ACD, 50%). Both methods suggest Eurasia (A) as the ancestral area for Allieae (Clade I; node 2). S-DIVA suggests Africa + South America (CD) as the most probable ancestral area for clade II (node 3), which includes the remaining tribes of Allioideae, whereas BBM indicated Africa (C) with 87% marginal probability. BBM and S-DIVA reconstructions both suggest that South America (D) is the most probable ancestral area for the node of Gilliesieae–Leucocoryneae (node 4).

## Discussion

### Chloroplast genome evolution in Allioideae

The newly sequenced chloroplast genome revealed a highly conserved genome structure in terms of GC content and gene composition and order among Allioideae and related taxa (Table 1 and Fig. 1). In comparison to GC content of *Amborella trichopoda* (38.34%), *Nicotiana tabacum* (37.85%), and *Oryza sativa* (39%), those of Allioideae exhibited a lower percentage, especially members of Allieae (generally ≤ 37.1%). However, only some representatives of over 1000 species in Alliodeae were used in this study. Therefore, a larger number of Allioideae samples is needed to clarify the fluctuation of GC content which contributed to RNA editing and stability of genome structure^23–25^. Gene content varied among species due to pseudogenization and loss of genes in some Allioideae (i.e., Tulbaghieae, Gillesieae, and Allieae; Table 1). The size of chloroplast genome was affected by the reduction and expansion of IR regions and gene loss and duplication^23^. Among Allioideae species, chloroplast genome size fluctuation was caused by pseudogenization and loss of genes (Table 1). For example, the smallest cpDNA in Allioideae was found in *Allium paradoxum* of which three and nine genes were lost and pseudogenized, respectively. Further observation on the gene loss and pseudogenization revealed that the gene loss and pseudogenization are not corresponded to the recognized clades indicating parallel evolution of these events in *Allium* (Table S5). For example, the loss of *infA* was recorded in representative species of three evolutionary lines in *Allium*. A similar trend was found in the pseudogenization of *rps2* of which the intact sequences were also recorded (Table S5). In monocots, the parallel loss or pseudogenization of genes has been reported. For instance, in Liliales representatives of both photosynthetic and mycoheterotrophic groups show the gene loss and pseudogenization of *rps16, infA*, and *cemA*^26^. Previously, the loss of *infA* was surveyed in angiosperms, revealing that the loss of *infA* from cpDNA can be mitigated by *infA* in the nuclear genome^27^. Various gene deletions have been reported in *Allium* (section *Daghestanica*)^21^. However, the mechanism leading to and outcomes of these events have not been studied in Allioideae species. In the present study, the sequence of the lost gene was not found in the current raw NGS data, suggesting that these genes were not transferred to nuclear or mitochondrial genomes. However, to confirm the final destination of the lost genes, the NGS data of nuclear and mitochondrial genomes among *Allium* species should be generated. Additionally, only 13 out of over 800 species of *Allium* were examined in the present study; therefore, further studies that cover all members of *Allium* should be conducted to provide a comprehensive understanding of the evolution of gene loss and pseudogenization in Allieae and related taxa.

Aside from the loss and pseudogenization of genes, which affect genome size, the expansion and contraction of IR regions resulted in differing junctions among LSC-IR-SSC regions and thus caused length variations in the cpDNA of Allioideae (Table 1). Previously, Wang et al.^28^ described different junction types in monocot species, ranging from *trnH-GUG* to *rpl22*. The LSC-IR junctions of basal angiosperms and monocots were also reported and divided into five types^26^. In the present study, the LSC-IR junction varied from *trnH-GUG* (type II, *Nothoscordum bonariense*) to *rpl22* (type IV, most of *Allium*; Table 1). Notably, type III of LSC-IR junction (located in the IGS between *rps19-rpl22*) was found in *Allium monanthum* (Table 1), suggesting high variability of this boundary in Allioideae. Similar to the LSC-IR junction, the SSC-IR border feature is variable among Allioideae species, which may show overlap, adjunction, or a gap between *ycf1* and *ndhF* as described in a previous study^26^ (Table 1). This junction is located within *ycf1* in cpDNA due to its long length. These characteristics of the LSC-IR-SSC junction have also been reported in other monocot groups^28,29^, suggesting similar patterns of structural variation among the cpDNA of monocots.

Analysis of nucleotide diversity and repeats in cpDNA sequences provides useful information for identifying molecular markers, reconstructing phylogenetic relationships, and exploring population genetics in angiosperms^30,31^. In this study, different SSRs were identified among Allioideae that may be useful for studies of molecular markers and population genetics of *Allium* in particular and Allioideae in general (Tables S3 and S4). Furthermore, eight hotspot regions of cpDNA were identified, which can be used in future studies of interspecies relationships among *Allium* species (Table S2). Another study on the complete plastomes of *Allium* revealed different genes with high nucleotide diversity (including *ndhK, ndhE, ndhA, rps16, psaI, rpl22, rpl32*, and *trnK-UUU*) in comparison with the present study^15^. These various findings might be caused by different taxon sampling and an insufficient number of samples among the studies. However, these results provided preliminary data on nucleotide diversity of plastomes for further studies that include all *Allium* taxa to identify the common hotspot regions across *Allium*.

### Phylogenetic relationships of Allioideae

Our MP and BI analyses consistently recovered Allioideae as sister to Amaryllidoideae (Fig. 2). This result is in line with previous molecular phylogenetic studies of Amaryllidaceae^5,6^. By contrast, Allioideae was found to be sister to a clade of Amaryllidoideae and Agapanthoideae inferred from data of nuclear ITS and plastid *matK*, *ndhF*, and *rbcL*^7^. Although Allioideae has superior ovary and solid style (vs. inferior ovary and hollow style in Amaryllidoideae), these characteristics are homoplasious in Asparagales^32^. Our phylogenomic study recovered Allieae as sister to the rest tribes of Allioideae (Fig. 2). The unique position of Allieae is also corroborated by having the synapomorphic, gynobasic style (vs. terminal in other tribes). Tulbaghieae, sister to Leucocoryneae-Gilliesieae, could be distinguished by the presence of corona in the flower. Moreover, the pseudogenization of *cemA* gene was only detected in Tulbaghieae. Gilliesieae and Leucocoryneae were strongly supported as sister in agreement with Sassone and Giussani^2^. This relationship is supported by several morphological characteristics such as terminal style position and absence of corona in the flower. In addition, both tribes were distributed in South America. In particular, Gilliesieae is restricted to Chile and Patagonia in Argentina, while Leucocoryneae is located in Argentina, Chile, Bolivia, Peru, Paraguay, Uruguay, and Brazil. Therefore, molecular phylogenetic relationships among tribes of Allioideae were supported by morphological and geographical evidence.

In the present study, *Allium* subg. *Melanocrommyum* and *A.* subg. *Cyathophora* were found to be non-monophyletic although 74 protein-coding genes were used (Fig. 2). Previous molecular phylogenetic studies of *Allium* revealed the non-monophyly of some subgenera^8,9,33^. For example, Li et al.^33^ reported paraphyly of the subgenera *Anguinum*, *Cepa*, *Allium*, *Reticulatobulbosa*, and *Polyprason* inferred from ITS and *rps16* sequences. Similarly, the monophyly of subgenera *Rhizirideum*, *Polyprason*, and *Cyathophora* was not corroborated by ITS and external transcribed spacer sequences^9^. Additionally, the phylogeny of *Allium* based on whole plastome sequences revealed the polyphyly of subgenera *Cepa* and *Polyprason*^8^. Albeit different molecular datasets have been used and resulted in non-monophyletic relationships, *Allium* species are always placed into three distinct clades, and accordingly, the hypothesis of three evolutionary lineages was proposed^10,33^. Among members of the genus *Allium*, the basic chromosome numbers are x = 7, 8, 9, 10, 14^7,34,35^. Additionally, natural interspecific hybridization has been reported in *Allium*^35^. The high chromosome diversity and hybridization in this genus might blur to propose a clear classification of *Allium*. Although 74 protein-coding genes were used in the present study, subgeneric relationships within *Allium* were not fully resolved. Therefore, further studies using more *Allium* samples and more molecular data (i.e., coding sequences in nuclear and mitochondrial genomes, and hotspot regions) should be conducted to provide better subgeneric classification of this complex genus of Allioideae and an explanation for the three distinct groups of *Allium*.

### Divergence time and biogeographic origins of Allioideae

Accurate estimation of divergence time in a certain plant group is important to understanding its biogeographic history. However, like most plant groups, the fossil record in Allioideae is sparse. When paleontological data are lacking, molecular estimates provide the only means for inferring the age of lineages, and multiple DNA regions are used to ensure the accuracy of divergence time estimates. Here, we used 74 cpDNA coding regions to estimate the divergence times of major clades in Allioideae. Previous studies also analyzed divergence times of Allioideae and resulted in different outcomes (Table S6). Our molecular dating analysis suggests that Allioideae diverged from its sister clade in the early Eocene (mean = 47.7 mya; 95% HPD = 40.8–56.5 mya). Similar divergence time of Allioideae (41.9 mya, 95% HDP = 34.5–47.6 mya) was estimated based on 48 shared chloroplast genes among 19 monocots families^8^. The diversification of Allioideae, which resulted in the formation of two major lineages, is estimated to have occurred in the middle Eocene (40.1 mya, 95% HPD = 28.5–55.3 mya; node 1 in Fig. 3). This estimate of the crown age of Allioideae is similar to that obtained in a previous research (37.0 mya, 95% HPD = 27.8–44.5 mya)^6^. This result is also supported by the fossil genus *Paleoallium*, which is similar to extant *Allium*, recently reported during the Eocene^36^. Thus, we believe that this is the most reliable estimate of the divergence time for Allioideae to date. However, Costa et al.^7^ presented an older divergence time of Allioideae (Table S6). In particular, Allioideae diverged in the Paleocene (63.2 mya, 95% HDP = 67.5–53.7 mya) followed by splits of Allieae (52.2 mya, 95% HDP = 58.1–44.4 mya) and Tulbaghieae and Gilliesieae (54.1 mya, 95% HDP = 65.1–37.11 mya)^7^. In comparison to the results of the current study, the older times might be caused by different sequence data matrix (four loci of which missing data were accounted for 20.5% of the matrix), and different calibration points (fossil leaf of Amaryllidaceae)^7^.

The species in four tribes of Allioideae distributed discontinuously, with complete separation between the Northern and Southern Hemispheres (Allieae, Eurasia, and North America; Tulbaghieae, Africa; Gilliesieae and Leucocoryneae, South America). In contrast to Dubouzet and Shinoda^37^, who suggested that the major lineages of Allioideae originated in the Northern Hemisphere, our biogeographic reconstructions based on BBM analysis suggest that this subfamily originated in Africa with high marginal probability, while S-DIVA suggests Eurasia + Africa or Eurasia + Africa + South America as the origin of Allioideae (Fig. 4, Table 2). The deepest branches of the topology originate in Africa, including the sister groups of subfamilies Amaryllidoideae and Agapanthoideae. Moreover, the age of the crown node of clade II (mean = 25.3 mya), which includes Tulbaghieae, Gilliesieae, and Leucocoryneae from the Southern Hemisphere, is older than that of Allieae (mean = 21.3 mya) from the Northern Hemisphere (Fig. 3). The initial diversification of Allioideae likely occurred due to climatic conditions. During the late Paleocene and early Eocene, a warming period occurred, producing a pronounced climate optimum that favored the diversification of major Allioideae lineages in Africa. The ancestor of Allioideae is believed to have originated in Africa, with the Allieae lineage then migrating towards warmer areas of the Northern Hemisphere when the global climate shifted to cooler conditions around 50–34 mya^38^. Dispersal from Africa to Europe is common among land plants with disjunct distributions in both regions^39^.

The ancestral range of the crown node of clade II, which includes Tulbaghieae, Gilliesieae, and Leucocoryneae, is in Africa according to our BBM analysis (Fig. 4 and Table 2). Two mechanisms have been proposed to explain the intercontinental distribution of this clade in the Southern Hemisphere, attributing it to either dispersal or vicariance (continental drift). We observed disjunct populations in Africa and South America. Our age estimate for the divergence of these two regions is 25.3 mya, followed by diversification approximately 16.5 mya and the subsequent emergence of the monophyletic Gilliesieae–Leucocoryneae lineage in South America. Thus, continental drift does not appear to have played a role in the disjunct distribution of the Allioideae species in Africa and South America, as the great southern continent of Gondwanaland is thought to have broken up in the early Cretaceous. The possibility of biological exchange between Africa and South America since the late Oligocene occurred too recent to support a vicariance explanation based on continental drift. Instead, long-distance dispersal may explain the intercontinental distribution of African and South American Allioideae species. Similar origins have been postulated for Caricaceae^40^ and Canellaceae^41^. The latest study on the biogeography of Allioideae suggested an “Out-of-India” hypothesis for the colonization of Allieae in the northern hemisphere from India tectonic plate^7^. However, the absence of Allieae species in India questioned the reliability of “Out-of-India” hypothesis although the authors demonstrated that aridification during the collision of India and Eurasia caused the extinction of *Allium* in India.

The present study presents the most detailed molecular phylogenetic and biogeographic information available to date for Allioideae and illustrates the need to investigate relationships at the tribe level more thoroughly, especially Gilliesieae–Leucocoryneae. Givnish et al.^42^ recently suggested an “out of Gondwana” origin for Liliales and emphasized the importance of vicariance in the ancient past for determining its current distribution. However, the biogeographic origin and their distribution pattern of Asparagales in the Southern Hemisphere have not yet been addressed. Thus, future works should include additional sampling to establish the biogeographic history of Asparagales in Southeast Asia, India, South America, Australia, and Africa.

## Conclusions

This study provided new data on the evolution of chloroplast genomes in Allioideae. Specifically, there were parallel events of gene loss (*infA, rps16, ndhF, ndhG,* and *rpl22*) and pseudogenization (i.e., *rps2, ycf15, rps16* and *matK*) across Allieae despite the division of *Allium* into evolutionary lines. The phylogeny inferred from 74 protein-coding genes revealed the monophyly of tribes in Allioideae; however, the subgenera classification of *Allium* was polyphyletic, suggesting further studies on phylogeny of *Allium* with more samples and molecular data (i.e., single copy genes in nuclear and mitochondrial genomes and non-coding regions). Divergence time estimation and biogeographic analysis resulted in the origin from Africa in the Eocene of Allioideae species of which the expansion to the northern hemisphere may infer from long-distance dispersal.

## Materials and methods

### Taxon sampling, DNA extraction, genome assembly, and annotation

Allioideae samples were collected from various sources (Table S7). Samples were dried with silica gel and used for extraction of total genomic DNA with a modified 2 × cetyltrimethylammonium bromide (CTAB) method^43^. High-quality DNA samples (> 200 ng/ul) were applied to NGS using the MiSeq sequencing platform with Miseq Reagent Kit v3 following manufacturer’s instruction (Illumina, Korea). The raw reads (2 × 300 bp paired-end reads) obtained were trimmed to remove regions with error probabilities greater than 0.01% per base using Geneious v.7.1.9^44^. Also, the adapter sequences were removed using the function “Trims Ends” of Geneious v.7.1.9. The paired-end reads (300 bp) were assembled using the reference chloroplast genomes of *Allium cepa* (GenBank no. KM088013), *Allium obliquum* (GenBank no. NC037199), *Allium sativum* (GenBank no. NC031829), *Allium ursinum* (GenBank no. MH157875), and *Allium victorialis* (GenBank no. MF687749) based on minimum similarity of 95% to the reference. Then, the isolated reads were subjected to de novo assembly in Geneious to complete the chloroplast genome sequences. The number of total reads, number of assembled reads, and coverage are summarised in Table S7 (over 15x). To confirm the newly completed sequences of *Allium* chloroplast genome, NOVOPlasty was used following the manual instructions^45^. In the case of having gaps during the assembly process, specific primer pairs were designed using Primer3 and the PCR products were sequenced using Sanger method to cover the gaps^46^. The newly completed chloroplast genome sequences were annotated using previously published *Allium* cpDNA as listed above with Geneious. Then, the protein-coding regions were checked and manually adjusted to include a start codon at the beginning and a stop codon at the end of the region. The tRNA sequences were confirmed using tRNAScan-SE^47^. A circular chloroplast genome map was obtained using the OGDraw program^48^.

### Comparative genomic analyses in Allioideae

The new complete cpDNA sequences of Allioideae species were used along with published cpDNA from NCBI (including *Allium cepa* [GenBank no. KM088013], *A. obliquum* [GenBank no. NC037199], *A. sativum* [GenBank no. NC031829], *A. ursinum* [GenBank no. MH157875], and *A. victorialis* [GenBank no. MF687749]) for comparative analysis (Table S7). The DNASP 5.0 program was used to calculate the nucleotide diversity (Pi values) of noncoding and coding cpDNA regions among Allioideae species^49^. The REPuter program was used to identify repeats in the cpDNA of Allioideae with a minimum length of 19 bp^50^. The Phobos program embedded in Geneious was used to identify simple single repeats, including mono-, di-, tri-, tetra-, penta-, and hexa-nucleotides with repeated numbers of 10, 5, 4, 3, 3, and 3, respectively [http://www.rub.de/ecoevo/cm/cm_phobos.htm].

### Phylogenetic analysis

Twenty-eight species were subjected to phylogenetic analysis, including Allioideae (21 species), Amaryllidoideae (5), and Agapanthoideae (1) within Amaryllidaceae*.* Within Allioideae, all four tribes (Allieae [18 species], Gilliesieae [1], Leucocoryneae [1], and Tulbaghieae [1]) recognized in the most recent accounts of the subfamily were sampled. For rooting, five species of Asparagaceae, Xanthorrhoeaceae, and Iridaceae were included based on previous phylogenetic studies^6^. Taxa sampled, voucher information, and GenBank accession numbers for the cp genome data are listed in Table S4. Among 80 coding genes in the chloroplast genome, six genes (*rpl22, infA, ycf15, rps2, rps16*, and *accD*) were excluded from the data matrix due to pseudogenization and loss events. Thus, the phylogenetic analyses were done on a dataset of 74 coding genes of the cp genome. Multiple-sequence alignment was performed using MAFFT v.6^51^ with the default alignment parameters. Gaps were treated as missing data.

Phylogenetic reconstructions based on the combined sequences of 74 coding genes were performed using the maximum parsimony (MP) method in the program PAUP^*^ 4.0b10^52^. All characters and character states were weighted equally and unordered. The most parsimonious trees were identified with a heuristic algorithm comprising tree bisection-reconnection, branch swapping, the MULPARS function, and the alternative character state. Bootstrap analyses (1000 pseudoreplicates) were conducted to examine the relative level of support (BP) for individual clades on each of the resulting cladograms.

Phylogenetic analysis of the combined cpDNA dataset was also conducted using Bayesian inference (BI) in MrBayes v.3.12^53^. Applying the Akaike information criterion, jModelTest v.2.1.7^54^ assigned the GTR + I + Г model of molecular evolution to the combined dataset. Four MCMC chains were run simultaneously and sampled every 1000 generations for a total of 20 million generations. We plotted the log-likelihood scores of sample points against generation time using Tracer v.1.5; this ensured that stationarity was achieved after the first 2 million generations by determining whether the log-likelihood values of the sample points reached a stable equilibrium. In addition, we used the AWTY graphical system^55^ to compare split frequencies among runs and plot the cumulative split frequencies to ensure that stationarity was reached. The first 1000 (10%) sample trees from each run were discarded (representing burn-in), as determined using Tracer v.1.5. A maximum a posteriori tree was constructed by summarising the remaining trees from parallel runs into a majority-rule consensus tree, yielding posterior probability (PP) values for each clade.

### Molecular dating analysis

To estimate the divergence times of tribes in Allioideae, we used BEAST v.1.8^56^ based on 74 cpDNA coding regions. The BEAUti interface was used to generate input files for BEAST, in which the GTR + I + Г model, Yule speciation tree prior, and uncorrelated lognormal molecular clock model were applied. Two runs of 200 million generations were set for the MCMC chains, sampling every 1000 generations. Convergence of the stationary distribution was checked through visual inspection of the plotted posterior estimates using Tracer v.1.6. After discarding the first 20,000 (10%) trees as burn-in, the samples were summarised in a maximum clade credibility tree in TreeAnnotator v.1.6.1 using a PP limit of 0.50 and summarising the mean node heights. The mean and 95% HPD of each age estimate were obtained from the combined outputs using Tracer. The results were visualized using Figtree v.1.4.2 [http://tree.bio.ed.ac.uk/software/figtree/].

Age calibration was constrained to the phylogeny of Allioideae and its close relatives. The crown node (C1 in Fig. 3) of *Yucca*-*Hosta* was constrained with a uniform distribution from 20.7 to 37.5 mya following McKain et al.^57^, who estimated the divergence time of Agavoideae using 69 cpDNA coding genes. Three further calibration processes were implemented, as uniform distribution from 50.0 to 67.4 mya for the stem group of Amaryllidaceae (C2); from 42.0 to 61.7 mya for the crown group of Amaryllidaceae (C3); and from 38.1 to 56.5 mya for the stem node of Allioideae (C4).

### Ancestral area reconstruction

Biogeographic data for species within Allioideae were compiled from their distributions described in the literature and herbarium specimens. The distribution range of Allioideae species and outgroups was divided into five areas: (A) Eurasia, (B) North America, (C) Africa, (D) South America, and (E) Australia. We coded each species based on the entire range of the species regardless of the sample’s biogeographic source. Ancestral area reconstruction and estimation of spatial patterns of geographic diversification within Allioideae were inferred using the BBM and S-DIVA as implemented in RASP v.2.1b (Reconstruct Ancestral State in Phylogenies, formerly S-DIVA)^58^. The BBM was run using the fixed state frequencies model (Jukes-Cantor) with equal among-site rate variations over two million generations, 10 chains each, and two parallel runs. In S-DIVA, the frequencies of ancestral ranges at a given node in ancestral reconstructions are averaged over all trees. For these analyses, we used all post burn-in trees obtained from BEAST analysis. The consensus tree used to map the ancestral distribution of each node was obtained using the Compute Condense option in RASP from stored trees. The maximum number of ancestral areas was set to five.

## Supplementary Information

Supplementary Tables.
