# Supplementary material for: Complete chloroplast genomes shed light on phylogenetic relationships, divergence time, and biogeography of Allioideae (Amaryllidaceae)
Source: Sci Rep. 2021 Feb 5;11:3262. doi: 10.1038/s41598-021-82692-5 (PMC7865063; doi:10.1038/s41598-021-82692-5)
Supplement: Supplementary file 1 — Supplementary Tables. [file 41598_2021_82692_MOESM1_ESM.docx]

**Complete chloroplast genomes shed light on phylogenetic relationships, divergence time, and biogeography of Allioideae (Amaryllidaceae)**

Ju Namgung ^§1^, Hoang Dang Khoa Do^§1,3^, Changkyun Kim^1^, Hyeok Jae Choi^2^, Joo-Hwan Kim^1^*

^1^ Department of Life Science, Gachon University, Seongnam, Republic of Korea 13120

^2^ Department of Biology and Chemistry, Changwon National University

^3^ Nguyen Tat Thanh Hi-Tech Institute, Nguyen Tat Thanh University, Ho Chi Minh City, Vietnam

§: These authors contributed equally in this study.

Supplementary Tables: **Table S1**: The gene content in the chloroplast genome of *Allium* and related taxa. **Table S2**: The nucleotide diversity (Pi (π) value) of coding and non-coding regions among Allioideae species. **Table S3**: The Feature of repeats in *Allium* species. **Table S4**: The distribution of SSRs among Allioideae species. **Table S5**. Gene loss and pseudogenization in Allium and related taxa of Allioideae. **Table S6**: Comparison of divergence times of Allioideae and its tribes in different studies.**Table S7**: List of species used for comparative genomics.

Table S1: The gene content in the chloroplast genome of *Allium* and related taxa

| Group of gene | | Name of gene(common) |
| --- | --- | --- |
| RNA genes | Ribosomal RNAs | *rrn4.5(x2), rrn5(x2), rrn16(x2), rrn23(x2)* |
|  | Transfer RNAs | *trnA-UGCa(x2), trnC-GCA, trnD-GUC, trnE-UUC, trnF-GAA, trnfM-CAU, trnG-GCC, trnG-UCCa, trnH-GUG, trnI-CAU(x2), trnI-GAUa(x2), trnK-UUUa, trnL-CAA(x2), trnL-UAAa, trnL-UAG, trnM-CAU, trnN-GUU(x2), trnP-UGG, trnQ-UUG, trnR-ACG(x2), trnR-UCU, trnS-GCU, trnS-GGA, trnS-UGA, trnT-GGU, trnT-UGU, trnV-GAC(x2), trnV-UACa, trnW-CCA, trnY-GUA* |
| Protein genes | Photosystem I | *psaA, psaB, psaC, psaI, psaJ* |
|  | Photosystem II | *psbA, psbB, psbC, psbD, psbE, psbF, psbH, psbI, psbJ, psbK, psbL, psbM, psbN, psbT, psbZ* |
|  | Cytochrome | *petA, petB, petD, petG, petL, petN* |
|  | ATP synthase | *atpA, atpB, atpE, atpFa, atpH, atpI* |
|  | Rubisco | *rbcL* |
|  | NADH dehydrogenase | *ndhAa,ndhBa(x2),ndhC,ndhD,ndhE, ndhF, ndhG, ndhH, ndhI, ndhJ, ndhK* |
|  | ATP-dependent protease subunit P | *clpPa* |
|  | Chloroplast envelope membrane protein | *cemA^z^* |
| Ribosomal proteins | large units | *rpl2a(x2), rpl14, rpl16, rpl20, rpl22^x^, rpl23^z^(x2), rpl32, rpl33, rpl36* |
|  | small units | *rps2^z^, rps3, rps4, rps7(x2), rps8, rps11, rps12a(x2), rps14, rps15, rps16^z^, rps18, rps19(x2)* |
| Transcription | RNA polymerase | *rpoA, rpoB, rpoC1a,rpoC2* |
| /translation | Initiation factor | *infA^zx^* |
|  | Miscellaneous proteins | *accD^z^, ccsA, matK^z^* |
|  | Hypothetical proteins & Conserved reading frame | *ycf1^z^, ycf2^z^(x2), ycf3, ycf4, ycf15^z^* |
| a: gene has intron; x2: gene has two copies; x : Loss; z : pseudogenization | | |

Table S2: The nucleotide diversity (Pi (π) value) of coding and non-coding regions among Allioideae species.

| **No** | **Region** | **Allioideae** | | | ***Allium*** | | |
| --- | --- | --- | --- | --- | --- | --- | --- |
|  |  | **Length (bp)** | **Pi value** | **SD value** | **Length (bp)** | **Pi value** | **SD value** |
| 1 | ***psbA* upstream** | **240** | **0,09463** | **0,00841** | **225** | **0,08718** | **0,00849** |
| 2 | *psbA-trnK* | 310 | 0,02908 | 0,00576 | 305 | 0,02883 | 0,00458 |
| 3 | *trnK-matK* | 386 | 0,03216 | 0,00685 | 373 | 0,0256 | 0,00263 |
| 4 | *matk-trnK* | 942 | 0,03826 | 0,00585 | 880 | 0,02765 | 0,00266 |
| 5 | *trnK-rps16* | 705 | 0,06926 | 0,00914 | 642 | 0,0493 | 0,00429 |
| 6 | *rps16 intron* | 1039 | 0,0429 | 0,00613 | 984 | 0,03789 | 0,00277 |
| 7 | ***rps16-trnQ-UUG*** | **1798** | **0,09551** | **0,02371** | 1665 | 0,04659 | 0,00559 |
| 8 | *trnQ-UUG-psbK* | 411 | 0,04557 | 0,00809 | 371 | 0,02947 | 0,00338 |
| 9 | *psbK-psbI* | 514 | 0,05007 | 0,00642 | 498 | 0,04207 | 0,00434 |
| 10 | *psbI-trnS-GCU* | 121 | 0,08187 | 0,001692 | 40 | 0,05917 | 0,0107 |
| 11 | *trnS-GCU-trnG-UCC* | 1739 | 0,06426 | 0,0116 | 1614 | 0,0406 | 0,00304 |
| 12 | *trnG-UCC-trnR-UCU* | 163 | 0,06644 | 0,00886 | 158 | 0,05131 | 0,00613 |
| 13 | *trnR-UCU-atpA* | 117 | 0,06471 | 0,00838 | 130 | 0,05525 | 0,00768 |
| 14 | *atpA-atpF* | 81 | 0,02628 | 0,00518 | 82 | 0,01392 | 0,00301 |
| 15 | *atpF intron1* | 1045 | 0,03106 | 0,00436 | 955 | 0,02206 | 0,00222 |
| 16 | *atpF-atpH* | 595 | 0,04307 | 0,00711 | 433 | 0,02829 | 0,00235 |
| 17 | *atpH-atpI* | 575 | 0,04378 | 0,00708 | 550 | 0,02987 | 0,00406 |
| 18 | *atpI-rps2* | 363 | 0,03676 | 0,00577 | 239 | 0,02454 | 0,00244 |
| 19 | *rps2-rpoC2* | 195 | 0,05832 | 0,01132 | 184 | 0,03561 | 0,0072 |
| 20 | *rpoC2-rpoC1* | 202 | 0,04355 | 0,00855 | 192 | 0,02561 | 0,00454 |
| 21 | *rpoC1 intron1* | 694 | 0,03629 | 0,00606 | 813 | 0,02342 | 0,00261 |
| 22 | *rpoC1-rpoB* | 26 | 0,01722 | 0,00575 | 26 | 0,01559 | 0,00558 |
| 23 | *rpoB-trnC-GCA* | 1475 | 0,05405 | 0,00787 | 1415 | 0,03725 | 0,00323 |
| 24 | *trnC-GCA-petN* | 1358 | 0,05094 | 0,00807 | 1016 | 0,03226 | 0,00335 |
| 25 | *petN-psbM* | 1183 | 0,06172 | 0,00918 | 982 | 0,04635 | 0,00341 |
| 26 | *psbM-trnD-GUC* | 1268 | 0,05926 | 0,0091 | 1189 | 0,03955 | 0,00292 |
| 27 | *trnD-GUC-trnY-GUA* | 413 | 0,05482 | 0,000723 | 367 | 0,0343 | 0,00409 |
| 28 | *trnY-GUA-trnE-UUC* | 59 | 0,03269 | 0,00779 | 59 | 0,01784 | 0,00421 |
| 29 | *trnE-UUC-trnT-Ggu* | 531 | 0,05959 | 0,01137 | 400 | 0,03521 | 0,00305 |
| 30 | *trnT-GGU-psbD* | 1380 | 0,05722 | 0,00892 | 1311 | 0,04002 | 0,00287 |
| 31 | *psbC-trnS-UGA* | 152 | 0,07038 | 0,00904 | 148 | 0,05162 | 0,00556 |
| 32 | *trnS-UGA-psbZ* | 435 | 0,0399 | 0,00676 | 415 | 0,03042 | 0,00353 |
| 33 | *psbZ-trnG-GCC* | 335 | 0,05224 | 0,00506 | 321 | 0,04024 | 0,00316 |
| 34 | *trnG-GCC-trnfM-CAU* | 386 | 0,02169 | 0,00615 | 367 | 0,01924 | 0,00666 |
| 35 | *trnfM-CAU-rps14* | 163 | 0,03468 | 0,0045 | 162 | 0,0293 | 0,00417 |
| 36 | *rps14-psaB* | 145 | 0,0312 | 0,00884 | 138 | 0,01631 | 0,00276 |
| 37 | *psaB-psaA* | 25 | 0,00381 | 0,00337 | 25 | 0 | 0 |
| 38 | *psaA-ycf3* | 731 | 0,03571 | 0,00492 | 737 | 0,02608 | 0,00222 |
| 39 | *ycf3 intron1* | 784 | 0,02695 | 0,00463 | 763 | 0,01719 | 0,00124 |
| 40 | *ycf3 intron2* | 758 | 0,02851 | 0,00368 | 735 | 0,02129 | 0,00197 |
| 41 | *ycf3-trnS-GGA* | 608 | 0,04475 | 0,0089 | 537 | 0,02675 | 0,00202 |
| 42 | *trns-GGA-rps4* | 316 | 0,05938 | 0,0084 | 300 | 0,04163 | 0,00375 |
| 43 | *rps4-trnT-UGU* | 412 | 0,04499 | 0,00772 | 407 | 0,02886 | 0,00261 |
| 44 | *trnT-UGU- trnL-UAA* | 1535 | 0,06228 | 0,01325 | 1450 | 0,02984 | 0,00257 |
| 45 | *trnL-UAA-trnF-GAA* | 465 | 0,07947 | 0,02061 | 336 | 0,04042 | 0,00356 |
| 46 | *trnF-GAA-ndhJ* | 787 | 0,05206 | 0,01062 | 769 | 0,0303 | 0,00244 |
| 47 | *ndhJ-ndhK* | 120 | 0,04899 | 0,00629 | 120 | 0,03753 | 0,0035 |
| 48 | *ndhC-trnV-UAC* | 2272 | 0,08507 | 0,02155 | 625 | 0,04587 | 0,0026 |
| 49 | *trnV-UAC-trnM-CAU* | 164 | 0,04439 | 0,00669 | 159 | 0,03082 | 0,00336 |
| 50 | *trnM-CAU-atpE* | 185 | 0,05788 | 0,01049 | 179 | 0,05257 | 0,01117 |
| 51 | *atpB-rbcL* | 918 | 0,03815 | 0,00548 | 854 | 0,0267 | 0,00192 |
| 52 | *rbcL-accD* | 905 | 0,04542 | 0,00771 | 905 | 0,02627 | 0,0027 |
| 53 | *accD-psaI* | 575 | 0,04775 | 0,0079 | 585 | 0,03305 | 0,0043 |
| 54 | *psbI-ycf4* | 421 | 0,03769 | 0,00731 | 384 | 0,02296 | 0,00384 |
| 55 | *ycf4-cemA* | 478 | 0,03501 | 0,00735 | 425 | 0,01929 | 0,00339 |
| 56 | *cemA-petA* | 252 | 0,04866 | 0,00609 | 242 | 0,04113 | 0,00409 |
| 57 | *petA-psbJ* | 1399 | 0,05529 | 0,00743 | 1338 | 0,04254 | 0,00312 |
| 58 | *psbJ-psbL* | 126 | 0,0203 | 0,00369 | 125 | 0,01646 | 0,00361 |
| 59 | *psbL-psbF* | 22 | 0,01299 | 0,0063 | 22 | 0,00505 | 0,00438 |
| 60 | *psbF-psbE* | 17 | 0,0056 | 0,00496 | 28 | 0,00654 | 0,00567 |
| 61 | *psbE-petL* | 1096 | 0,04381 | 0,00664 | 1076 | 0,02827 | 0,024 |
| 62 | *petL-petG* | 271 | 0,04414 | 0,00733 | 247 | 0,02767 | 0,00374 |
| 63 | ***petG-trnW-CCA*** | **144** | **0,0955** | **0,0095** | **140** | **0,07957** | **0,00671** |
| 64 | *trnW-CCA-trnP-UGG* | 186 | 0,07561 | 0,01309 | 182 | 0,04552 | 0,00531 |
| 65 | *trnP-UGG-psaJ* | 434 | 0,03704 | 0,00607 | 436 | 0,02861 | 0,00306 |
| 66 | *psaJ-rpl33* | 745 | 0,05326 | 0,00621 | 470 | 0,03816 | 0,00307 |
| 67 | *rpl33-rps18* | 196 | 0,05327 | 0,00903 | 177 | 0,03297 | 0,00518 |
| 68 | *rps18-rpl20* | 360 | 0,05407 | 0,00939 | 351 | 0,03884 | 0,0075 |
| 69 | *rpl20-rps12* | 889 | 0,02796 | 0,00546 | 849 | 0,01877 | 0,00217 |
| 70 | *rps12-clpP* | 132 | 0,03372 | 0,00585 | 126 | 0,02245 | 0,00279 |
| 71 | *clpP intron1* | 715 | 0,03358 | 0,00491 | 689 | 0,02118 | 0,00182 |
| 72 | *clpP intron2* | 846 | 0,04337 | 0,00687 | 845 | 0,02812 | 0,00298 |
| 73 | *clpP-psbB* | 478 | 0,04068 | 0,00717 | 465 | 0,02573 | 0,00345 |
| 74 | *psbB-psbT* | 213 | 0,04229 | 0,0082 | 203 | 0,02548 | 0,00442 |
| 75 | *psbT-psbN* | 72 | 0,07364 | 0,01092 | 71 | 0,05615 | 0,0063 |
| 76 | *psbN-psbH* | 116 | 0,03007 | 0,00601 | 116 | 0,01796 | 0,00388 |
| 77 | *psbH-petB* | 125 | 0,04884 | 0,00833 | 121 | 0,03961 | 0,00526 |
| 78 | *petB intron1* | 862 | 0,0429 | 0,00622 | 859 | 0,03096 | 0,00276 |
| 79 | *petB-petD* | 255 | 0,0319 | 0,00684 | 258 | 0,01814 | 0,00279 |
| 80 | *petD intron1* | 787 | 0,02729 | 0,00444 | 771 | 0,01773 | 0,00173 |
| 81 | ***petD-rpoA*** | 179 | 0,07495 | 0,01081 | **146** | **0,06643** | **0,01098** |
| 82 | *rpoA-rps11* | 73 | 0,03144 | 0,00444 | 73 | 0,02677 | 0,00433 |
| 83 | *rps11-rpl36* | 132 | 0,03681 | 0,00482 | 132 | 0,0291 | 0,00343 |
| 84 | *rpl36-infA* | 160 | 0,04949 | 0,00712 | 160 | 0,04301 | 0,0068 |
| 85 | *infA-rps8* | 142 | 0,05372 | 0,00542 | 141 | 0,04666 | 0,00442 |
| 86 | *rps8-rpl14* | 309 | 0,0555 | 0,01141 | 290 | 0,03724 | 0,00317 |
| 87 | *rpl14-rpl16* | 147 | 0,04316 | 0,00676 | 137 | 0,032263 | 0,00354 |
| 88 | *rpl16 intron1* | 1375 | 0,04505 | 0,0065 | 1257 | 0,03113 | 0,00237 |
| 89 | *rpl16-rps3* | 256 | 0,07341 | 0,00996 | 243 | 0,05283 | 0,00894 |
| 90 | *rps3-rpl22* | 61 | 0,06723 | 0,01212 | 61 | 0,03012 | 0,00606 |
| 91 | *rpl22-rps19* | 55 | 0,0369 | 0,01498 | 50 | 0,0222 | 0,01046 |
| 92 | *rps19-trnH-GuG* | 146 | 0,00446 | 0,00192 | 142 | 0,00353 | 0,00178 |
| 93 | *trnH-GUG-rpl2* | 43 | 0,04917 | 0,00787 | 43 | 0,4164 | 0,01075 |
| 94 | *rpl2 intron1* | 671 | 0,0039 | 0,00093 | 662 | 0,0027 | 0,00071 |
| 95 | *rpl2-rpl23* | 89 | 0,01317 | 0,00419 | 81 | 0,00533 | 0,00267 |
| 96 | *rpl23-trnI-CAU* | 165 | 0,0077 | 0,00136 | 165 | 0,00792 | 0,00141 |
| 97 | *trnI-CAU-ycf2* | 91 | 0,00691 | 0,00353 | 165 | 0,00067 | 0,00058 |
| 98 | *ycf2-ycf15* | 115 | 0,00107 | 0,00095 | 81 | 0,00533 | 0,00267 |
| 99 | *ycf15-trnL-CAA* | 681 | 0,00492 | 0,00154 | 681 | 0,00237 | 0,00055 |
| 100 | *trnL-CAA-ndhB* | 595 | 0,0071 | 0,00122 | 575 | 0,00532 | 0,00066 |
| 101 | *ndhB intron* | 704 | 0,0058 | 0,0009 | 704 | 0,00478 | 0,00076 |
| 102 | *ndhB-rps7* | 322 | 0,00737 | 0,00161 | 319 | 0,0355 | 0,0008 |
| 103 | *rps7-rps12* | 58 | 0,02024 | 0,00598 | 58 | 0,0084 | 0,00147 |
| 104 | rps12 CDS2- rps12 CDS3 | 552 | 0,00591 | 0,00193 | 542 | 0,00164 | 0,0005 |
| 105 | *rps12-trnV-GAC* | 1944 | 0,01132 | 0,00275 | 1530 | 0,00548 | 0,00077 |
| 106 | *trnV-GAC-rrn16* | 231 | 0,01156 | 0,00296 | 231 | 0,0061 | 0,00131 |
| 107 | *rrn16-trnI-GAU* | 301 | 0,01075 | 0,00292 | 301 | 0,0049 | 0,00126 |
| 108 | *trnI-GAU-trnA-UGC* | 64 | 0,00298 | 0,00182 | 64 | 0,00174 | 0,00151 |
| 109 | *trnA-UGC-rrn23* | 150 | 0,01619 | 0,00245 | 150 | 0,01208 | 0,00204 |
| 110 | *rrn23-rrn4_5* | 99 | 0,00452 | 0,00187 | 99 | 0,00112 | 0,00097 |
| 111 | *rrn4_5_rrn5* | 231 | 0,01733 | 0,00346 | 230 | 0,0101 | 0,00141 |
| 112 | *rrn5-trnR-ACG* | 260 | 0,01036 | 0,00273 | 239 | 0,0047 | 0,00095 |
| 113 | *trnR-ACG-trnN-GUU* | 630 | 0,02018 | 0,00315 | 617 | 0,01633 | 0,0013 |
| 114 | *trnNGUU-ndhF* | 1475 | 0,01017 | 0,00239 | 1620 | 0,00532 | 0,00058 |
| 115 | *ndhF-rpl32* | 1375 | 0,06992 | 0,01138 | 1199 | 0,04442 | 0,00387 |
| 116 | ***rpl32-trnL-UAG*** | **1234** | **0,09359** | **0,01177** | **1044** | **0,06765** | **0,00486** |
| 117 | *trnL-UAG-ccsA* | 89 | 0,02901 | 0,00559 | 83 | 0,02182 | 0,00555 |
| 118 | *ccsA-ndhD* | 311 | 0,07872 | 0,0093 | 288 | 0,06539 | 0,00688 |
| 119 | *ndhD-psaC* | 126 | 0,04921 | 0,00605 | 126 | 0,03589 | 0,003 |
| 120 | *psaC-ndhE* | 562 | 0,06628 | 0,01127 | 540 | 0,04543 | 0,00709 |
| 121 | *ndhE-ndhG* | 205 | 0,06632 | 0,00827 | 165 | 0,04764 | 0,00481 |
| 122 | *ndhG-ndhI* | 449 | 0,08486 | 0,01126 | 418 | 0,06113 | 0,00447 |
| 123 | *ndhI-ndhA* | 88 | 0,04338 | 0,00866 | 88 | 0,0275 | 0,00587 |
| 124 | *ndhA intron* | 1257 | 0,04533 | 0,00624 | 1206 | 0,03317 | 0,00312 |
| 125 | *ndhH-rps15* | 149 | 0,06217 | 0,00618 | 130 | 0,05491 | 0,00399 |
| 126 | ***rps15-ycf1*** | **464** | **0,09956** | **0,301461** | **415** | **0,07296** | **0,0071** |
| 127 | *psbA* | 1062 | 0,01006 | 0,00165 | 1062 | 0,00704 | 0,00082 |
| 128 | ***matK*** | **1584** | **0,04527** | **0,00745** | 1581 | 0,02896 | 0,00222 |
| 129 | *rps16* | 262 | 0,03459 | 0,00597 | 262 | 0,02536 | 0,00485 |
| 130 | *trnQ-UUG* | 72 | 0,00747 | 0,00265 | 72 | 0,00309 | 0,00184 |
| 131 | *psbK* | 186 | 0,01608 | 0,00261 | 186 | 0,01233 | 0,00215 |
| 132 | *psbI* | 113 | 0,02701 | 0,00312 | 114 | 0,02438 | 0,00323 |
| 133 | *trnS-GCU* | 88 | 0,00963 | 0,0017 | 88 | 0,00928 | 0,00152 |
| 134 | *trnG-UCC* | 824 | 0,03504 | 0,00565 | 819 | 0,02434 | 0,00206 |
| 135 | *trnR-UCU* | 72 | 0,00265 | 0,00161 | 72 | 0,00154 | 0,00134 |
| 136 | *atpA* | 1524 | 0,02196 | 0,00364 | 1524 | 0,01393 | 0,0012 |
| 137 | *atpF* | 555 | 0,02147 | 0,00298 | 555 | 0,0154 | 0,00129 |
| 138 | *atpH* | 246 | 0,01812 | 0,00377 | 246 | 0,00972 | 0,00132 |
| 139 | *atpI* | 744 | 0,0193 | 0,0038 | 744 | 0,0108 | 0,00119 |
| 140 | ***rps2*** | 765 | 0,03336 | 0,00322 | **765** | **0,02961** | **0,00278** |
| 141 | *rpoC2* | 4222 | 0,02906 | 0,00478 | 4224 | 0,01886 | 0,00156 |
| 142 | *rpoC1* | 2052 | 0,01909 | 0,00016 | 2052 | 0,01262 | 0,00063 |
| 143 | *rpoB* | 3213 | 0,01974 | 0,00313 | 3213 | 0,01284 | 0,00106 |
| 144 | *trnC-GCA* | 77 | 0,00622 | 0,00208 | 71 | 0,00571 | 0,00204 |
| 145 | *petN* | 90 | 0,01153 | 0,00299 | 90 | 0,00581 | 0,00053 |
| 146 | *psbM* | 105 | 0,02014 | 0,00471 | 105 | 0,01239 | 0,00303 |
| 147 | *trnD-GUC* | 74 | 0,00257 | 0,00157 | 74 | 0,003 | 0,00179 |
| 148 | *trnY-GUA* | 84 | 0,0031 | 0,00133 | 83 | 0 | 0 |
| 149 | *trnE-UUC* | 73 | 0,00848 | 0,00129 | 73 | 0,00842 | 0,00175 |
| 150 | *trnT-GGU* | 72 | 0,00265 | 0,00161 | 72 | 0 | 0 |
| 151 | *psbD* | 1062 | 0,01462 | 0,00247 | 1062 | 0,00919 | 0,00079 |
| 152 | *psbC* | 1445 | 0,01491 | 0,00187 | 1445 | 0,01091 | 0,00083 |
| 153 | *trnS-UGA* | 88 | 0,01106 | 0,0031 | 87 | 0,00466 | 0,00167 |
| 154 | *psbZ* | 189 | 0,01058 | 0,0025 | 189 | 0,00664 | 0,00479 |
| 155 | *trnG-GCC* | 71 | 0,00134 | 0,00119 | 71 | 0 | 0 |
| 156 | *trnfM-CAU* | 74 | 0,00347 | 0,00149 | 74 | 0,00397 | 0,00161 |
| 157 | *rps14* | 303 | 0,02101 | 0,00446 | 303 | 0,01189 | 0,00216 |
| 158 | *psaB* | 2205 | 0,01244 | 0,00245 | 2205 | 0,00726 | 0,00092 |
| 159 | *psaA* | 2253 | 0,01376 | 0,00238 | 2253 | 0,00849 | 0,00061 |
| 160 | *ycf3* | 510 | 0,01716 | 0,00256 | 510 | 0,01177 | 0,00082 |
| 161 | *trnS-GGA* | 88 | 0,01093 | 0,00203 | 88 | 0,00728 | 0,00119 |
| 162 | *rps4* | 606 | 0,015 | 0,00281 | 606 | 0,00925 | 0,00132 |
| 163 | *trnT-UGU* | 73 | 0,00639 | 0,00103 | 73 | 0,00689 | 0,00088 |
| 164 | *trnL-UAA* | 626 | 0,01833 | 0,00392 | 401 | 0,01321 | 0,00282 |
| 165 | *trnF-GAA* | 70 | 0 | 0 | 70 | 0 | 0 |
| 166 | *ndhJ* | 477 | 0,013555 | 0,00236 | 477 | 0,00862 | 0,00078 |
| 167 | *ndhK* | 774 | 0,02308 | 0,00041 | 774 | 0,01454 | 0,00151 |
| 168 | *ndhC* | 363 | 0,01499 | 0,00296 | 363 | 0,00893 | 0,00108 |
| 169 | *trnV-UAC* | 682 | 0,0192 | 0,00307 | 679 | 0,01412 | 0,00215 |
| 170 | *trnM-CAU* | 81 | 0,00744 | 0,00268 | 73 | 0,00304 | 0,00181 |
| 171 | *atpE* | 422 | 0,02553 | 0,00442 | 405 | 0,01579 | 0,00142 |
| 172 | *atpB* | 1497 | 0,01708 | 0,00252 | 1497 | 0,0121 | 0,0014 |
| 173 | *rbcL* | 1482 | 0,01485 | 0,00276 | 1461 | 0,00901 | 0,00094 |
| 174 | *accD* | 1555 | 0,02755 | 0,00438 | 1515 | 0,02185 | 0,00208 |
| 175 | *psaI* | 112 | 0,02969 | 0,00331 | 111 | 0,02544 | 0,00289 |
| 176 | *ycf4* | 1034 | 0,01743 | 0,00357 | 555 | 0,01008 | 0,00128 |
| 177 | *cemA* | 696 | 0,02406 | 0,00391 | 692 | 0,01621 | 0,00177 |
| 178 | *petA* | 969 | 0,01838 | 0,00354 | 969 | 0,01068 | 0,00108 |
| 179 | *psbJ* | 123 | 0,01355 | 0,00281 | 123 | 0,00941 | 0,00242 |
| 180 | *psbL* | 117 | 0,0048 | 0,00181 | 117 | 0,00464 | 0,00195 |
| 181 | *psbF* | 120 | 0,02056 | 0,00313 | 120 | 0,01639 | 0,0024 |
| 182 | *psbE* | 252 | 0,00833 | 0,00151 | 252 | 0,00662 | 0,00137 |
| 183 | *petL* | 96 | 0,02525 | 0,00752 | 96 | 0,01028 | 0,00298 |
| 184 | *petG* | 114 | 0,01495 | 0,00387 | 114 | 0,0074 | 0,00198 |
| 185 | *trnW-CCA* | 74 | 0,00386 | 0,00187 | 74 | 0,0045 | 0,00212 |
| 186 | *trnP-UGG* | 74 | 0 | 0 | 74 | 0 | 0 |
| 187 | *psaJ* | 145 | 0,01846 | 0,00308 | 129 | 0,01297 | 0,00186 |
| 188 | *rpl33* | 201 | 0,02104 | 0,00455 | 201 | 0,0121 | 0,00266 |
| 189 | *rps18* | 318 | 0,01859 | 0,00303 | 306 | 0,1277 | 0,00187 |
| 190 | *rpl20* | 354 | 0,02526 | 0,00418 | 354 | 0,01665 | 0,00189 |
| 191 | *rps12* | 375 | 0,01161 | 0,00367 | 375 | 0,00376 | 0,00087 |
| 192 | *clpP* | 615 | 0,01558 | 0,00216 | 615 | 0,0118 | 0,00136 |
| 193 | *psbB* | 1527 | 0,01804 | 0,00321 | 1527 | 0,01103 | 0,00102 |
| 194 | *psbT* | 102 | 0,00717 | 0,00221 | 102 | 0,00327 | 0,00154 |
| 195 | *psbN* | 132 | 0,01436 | 0,00162 | 132 | 0,01277 | 0,00167 |
| 196 | *psbH* | 222 | 0,01587 | 0,00396 | 222 | 0,00733 | 0,00134 |
| 197 | *petB* | 663 | 0,01421 | 0,00101 | 663 | 0,00972 | 0,00117 |
| 198 | *petD* | 522 | 0,01358 | 0,00271 | 522 | 0,00815 | 0,0012 |
| 199 | *rpoA CDS* | 1020 | 0,02605 | 0,00377 | 1020 | 0,01827 | 0,00185 |
| 200 | *rps11 CDS* | 423 | 0,0217 | 0,00374 | 417 | 0,01331 | 0,00113 |
| 201 | *rpl36 CDS* | 114 | 0,01767 | 0,00314 | 114 | 0,01307 | 0,00209 |
| 202 | ***infA*** | 204 | 0 | 0 | **204** | **0,03806** | **0,00526** |
| 203 | *rps8* | 399 | 0,02629 | 0,00421 | 399 | 0,01727 | 0,00187 |
| 204 | *rpl14* | 369 | 0,01683 | 0,00356 | 369 | 0,00916 | 0,00127 |
| 205 | *rpl16* | 420 | 0,02221 | 0,00485 | 420 | 0,01205 | 0,00147 |
| 206 | *rps3* | 663 | 0,02664 | 0,00463 | 663 | 0,01661 | 0,00159 |
| 207 | ***rpl22*** | **411** | **0,04643** | **0,01849** | 402 | 0,1111 | 0,09636 |
| 208 | *rps19* | 279 | 0,00915 | 0,00184 | 279 | 0,0056 | 0,00085 |
| 209 | *trnH-GUG* | 74 | 0,00129 | 0,00114 | 7 | 0 | 0 |
| 210 | *rpl2* | 759 | 0,01001 | 0,00278 | 759 | 0,00406 | 0,00053 |
| 211 | *rpl23* | 282 | 0,00707 | 0,00142 | 282 | 0,0044 | 0,00093 |
| 212 | *trnI-CAU* | 74 | 0 | 0 | 74 | 0 | 0 |
| 213 | *ycf2* | 7015 | 0,00868 | 0,00122 | 6967 | 0,00612 | 0,00053 |
| 214 | *ycf15* | 232 | 0,01498 | 0,00195 | 232 | 0,01183 | 0,00155 |
| 215 | *trnL-CAA* | 81 | 0,00888 | 0,00122 | 81 | 0,00758 | 0,00132 |
| 216 | *ndhB* | 1533 | 0,00342 | 0,00073 | 1533 | 0,00207 | 0,00037 |
| 217 | *rps7* | 468 | 0,00439 | 0,0014 | 468 | 0,00138 | 0,00049 |
| 218 | *trnV-GAC* | 72 | 0 | 0 | 72 | 0 | 0 |
| 219 | *rrn16* | 1491 | 0,00089 | 0,00031 | 1491 | 0,00029 | 0,00014 |
| 220 | *trnI-GAU* | 1030 | 0,00383 | 0,00094 | 1021 | 0,00218 | 0,0003 |
| 221 | *trnA-UGC* | 916 | 0,00462 | 0,00086 | 910 | 0,00314 | 0,00054 |
| 222 | *rrn23* | 2810 | 0,00142 | 0,00022 | 2810 | 0,00107 | 0,00015 |
| 223 | *rrn4_5* | 103 | 0 | 0 | 104 | 0 | 0 |
| 224 | *rrn5* | 121 | 0,00299 | 0,00116 | 121 | 0,00173 | 0,00096 |
| 225 | *trnR-ACG* | 74 | 0 | 0 | 74 | 0 | 0 |
| 226 | *trnN-GUU* | 73 | 0,00352 | 0,00151 | 72 | 0 | 0 |
| 227 | ***ndhF*** | **2551** | **0,04227** | **0,00599** | **2387** | **0,02939** | **0,00227** |
| 228 | *rpl32* | 174 | 0,03703 | 0,00489 | 174 | 0,0284 | 0,00336 |
| 229 | *trnL-UAG* | 80 | 0,00226 | 0,0013 | 80 | 0,00261 | 0,00145 |
| 230 | *ccsA* | 972 | 0,03076 | 0,00514 | 972 | 0,02064 | 0,00172 |
| 231 | *ndhD* | 1503 | 0,03148 | 0,00449 | 1503 | 0,022 | 0,00169 |
| 232 | *psaC* | 247 | 0,01623 | 0,00309 | 246 | 0,01015 | 0,00149 |
| 233 | *ndhE* | 306 | 0,03139 | 0,0051 | 306 | 0,002083 | 0,00159 |
| 234 | *ndhG* | 531 | 0,03216 | 0,00471 | 531 | 0,02211 | 0,00212 |
| 235 | *ndhI* | 553 | 0,02267 | 0,0042 | 553 | 0,01397 | 0,00161 |
| 236 | *ndhA* | 1104 | 0,02728 | 0,00389 | 1104 | 0,01932 | 0,00141 |
| 237 | *ndhH* | 1182 | 0,02173 | 0,00339 | 1182 | 0,01451 | 0,00129 |
| 238 | *rps15* | 270 | 0,03014 | 0,00401 | 270 | 0,02263 | 0,00256 |
| 239 | ***ycf1*** | **5634** | **0,05133** | **0,00647** | **5421** | **0,03716** | **0,00293** |
| The bold numbers and gene names indicate the high variable regions in Allioideae | | | | | | | |

Table S3: The Feature of repeats in *Allium* species.

| No | Type | Length | Sequence | Location | Species |
| --- | --- | --- | --- | --- | --- |
| 1 | Forward | 28 | AATATATATATATATATATATAATTGAA | IGS (*trnT-GGU-psbD)* | only in *Allium koreanum* |
| 2 | Forward | 27 | GCCTATCCTCTCTAGTTCTGTTTGTAT | *atpF* intron | only in *Allium koreanum* |
| 3 | Forward | 25 | GGAGAGAGAGGGATTCGAACCCTCG | *trnS-GCU-trnS-UGA* | all examined species |
| 4 | Forward | 25 | ATTAGTTATTAATTAGTTATTTGGA | IGS (*psbZ-trnG-GCC)* | only in *Allium koreanum* |
| 5 | Forward | 24 | TATTCTTTTCCGATACATATAATA | IGS (*trnT-GGU-psbD*) | except *A. cernuum, A. karataviense, A. ochotense, A. sativum, A. spicatum, A. tricoccum,* and *A. tripedale* |
| 6 | Forward | 23 | AAATCTTTAGGAGGAATCAATGA | *trnI-CAU-ycf2* | all examined species |
| 7 | Forward | 23 | GATCACAAAAGACAAGAATTTGT | IGS (*trnT-GGU-psbD*) | Only in *A. koreanum* and *A. cepa* |
| 8 | Forward | 22 | GATGCGGGTTCGATTCCCGCTA | *trnG-UCC-trnG-GCC* | all examined species |
| 9 | Palindrome | 48 | AGTTTTTAAGAACTGTTTGAATTAAGATTCAAACAGTTCTTAAAAACT | IGS (*ccsA-ndhD*) | Only in *A. koreanum and A. obliquum* |
| 10 | Forward | 22 | ATATTGATGATAGTGACGATAT | *ycf2* | all species except *A. ursinum* |
| 11 | Forward | 21 | AAATAGATATATATAATATAG | IGS (*accD-psaI*) | only in *Allium koreanum* |
| 12 | Forward | 21 | ACATTAAAAAGTAGACTAAAA | IGS (*rpl32-trnL-UAG*) | only in *Allium koreanum* |
| 13 | Forward | 21 | TAATAGATACAAATATTAAAT | IGS (*accD-psaI*) | only in *A koreanum, A. cyathophorum, A. nigrum, A. obliquum, A. senescens, A. ursinum* |
| 14 | Forward | 20 | GACAGGATTTGAACCCGTGA | *trnfM-CAU-trnP-UGG* | all species except *A. ursinum* |
| 15 | Forward | 21 | TCTTTTTGTCCAAGTCACTTC | *ycf2* | all examined species |
| 16 | Forward | 20 | ACTTTATTTATTACAAATTA | IGS (*psaA-ycf3*) | only in *Allium koreanum* |
| 17 | Forward | 20 | TTGACATAAGAGATGTCATT | IGS (*trnN-GUU-ycf1*) | all species except *A. siculum* |
| 18 | Forward | 19 | TTTTTCCTAGTTTATTATA | IGS (*ndhF-rpl32*) | only in *A. koreanum&A. obliquum* |
| 19 | Forward | 19 | AATATAATATAAAAAATGA | IGS ((*trnS-GCU-trnG-UCC)*-(*ndhF-rpl32)*) | All species except *A. cyathophorum, Gilliesia graminea, Nothoscordum bonariense,* and *Tulbaghia violaceae.* |
| 20 | Forward | 19 | TGAAAATCCTCGTGTCACC | IGS (*trnL-UAA-trnF-GAA*)-*trnF-GAA* | all examined species except *Gilliesia graminea, Nothoscordum bonariense*, and *Tulbaghia violaceae.* |
| 21 | Forward | 19 | ATAGCTCAGTTGGTAGAGC | *trnF-GAA-trnA-UGC* | all examined species |

Table S4: The distribution of SSRs among Allioideae species.

| Type of repeat | Location | Length (bp) | | | | | | | | | | | | | | | | | | | | |
| --- | --- | --- | --- | --- | --- | --- | --- | --- | --- | --- | --- | --- | --- | --- | --- | --- | --- | --- | --- | --- | --- | --- |
|  |  | *A. koreanum* | *A. cepa* | *A. cernuum* | *A. cyathophorum* | *A. karataviense* | *A. monanthum* | *A. nerinifolium* | *A. nigrum* | *A. obliquum* | *A. ochotense* | *A. sativum* | *A. schoeprasum* | *A. senescense* | *A. spicatum* | *A. tricoccum* | *A. siculum* | *A. ursium* | *A. victorialis* | *Tulbaghia violaceae* | *Gilliesia graminea* | *Nothoscordum bonariense* |
| AT Repeat | IGS (*trnT-GGU-psbD)* | 20 | 6 | 6 | 6 | 6 | 6 | 2 | 4 | 4 | 6 | 6 | 6 | 4 | 6 | 6 | 6 | 6 | 6 | 4 | 0 | 4 |
| AT Repeat | IGS *(trnT-GGU-psbD)* | 20 | 12 | 8 | 26 | 16 | 8 | 8 | 12 | 12 | 10 | 12 | 12 | 12 | 6 | 12 | 8 | 8 | 10 | 2 | 0 | 2 |
| AT Repeat | IGS *(trnT-UGU-trnL-UAA)* | 14 | 10 | 8 | 12 | 8 | 10 | 8 | 10 | 10 | 8 | 6 | 10 | 10 | 8 | 8 | 12 | 16 | 8 | 0 | 0 | 0 |
| AT Repeat | IGS *(rpl32-trnL-UAG)* | 12 | 2 | 10 | 10 | 12 | 8 | 6 | 12 | 10 | 10 | 10 | 2 | 18 | 12 | 10 | 8 | 8 | 10 | 4 | 6 | 4 |
| AT Repeat | IGS *(rps4-trnT-UGU)* | 12 | 10 | 12 | 10 | 12 | 7 | 12 | 12 | 10 | 12 | 12 | 10 | 12 | 12 | 12 | 16 | 10 | 12 | 6 | 6 | 6 |
| AT Repeat | IGS *(trnS-UGA-psbZ)* | 12 | 12 | 14 | 14 | 8 | 10 | 8 | 10 | 12 | 8 | 12 | 12 | 12 | 8 | 8 | 12 | 12 | 8 | 16 | 8 | 8 |
| AT Repeat | IGS *(psaC-ndhE)* | 10 | 14 | 6 | 12 | 10 | 8 | 12 | 14 | 8 | 10 | 14 | 14 | 10 | 10 | 10 | 6 | 10 | 10 | 8 | 6 | 12 |
| AT Repeat | IGS *(rps18-rpl20)* | 10 | 10 | 6 | 8 | 6 | 6 | 6 | 10 | 10 | 4 | 16 | 10 | 10 | 6 | 6 | 8 | 6 | 6 | 4 | 4 | 6 |
| AT Repeat | IGS *(ycf4-cemA)* | 10 | 10 | 2 | 10 | 10 | 8 | 12 | 12 | 0 | 12 | 10 | 10 | 10 | 0 | 12 | 12 | 10 | 12 | 6 | 6 | 6 |
| AT Repeat | IGS *(rps4-trnT-UGU)* | 10 | 10 | 4 | 14 | 8 | 6 | 8 | 10 | 12 | 8 | 10 | 16 | 18 | 8 | 8 | 8 | 2 | 8 | 8 | 8 | 8 |
| AT Repeat | *rpoC2* | 10 | 10 | 10 | 10 | 10 | 10 | 10 | 10 | 10 | 10 | 10 | 10 | 10 | 10 | 10 | 10 | 10 | 10 | 10 | 10 | 10 |
| AG Repeat | *ycf2* | 10 | 10 | 10 | 10 | 10 | 10 | 10 | 10 | 10 | 10 | 10 | 10 | 10 | 10 | 10 | 10 | 10 | 10 | 10 | 10 | 8 |
| AG Repeat | *cemA* | 10 | 10 | 10 | 10 | 10 | 10 | 10 | 10 | 10 | 10 | 10 | 10 | 10 | 10 | 10 | 10 | 10 | 10 | 10 | 10 | 10 |
| AAT Repeat | IGS *(psbK-psbI)* | 12 | 12 | 12 | 12 | 12 | 12 | 12 | 12 | 12 | 12 | 12 | 12 | 12 | 0 | 12 | 9 | 7 | 12 | 6 | 6 | 6 |
| AAG Repeat | *clpP* exon 2 | 12 | 12 | 12 | 6 | 12 | 12 | 12 | 12 | 9 | 12 | 6 | 12 | 12 | 12 | 12 | 12 | 9 | 12 | 6 | 9 | 9 |
| A Repeat | *ndhA* intron | 10 | 13 | 10 | 8 | 9 | 8 | 11 | 9 | 10 | 9 | 8 | 11 | 10 | 10 | 10 | 10 | 11 | 9 | 10 | 4 | 4 |
| A Repeat | IGS *(psaC-ndhE)* | 10 | 8 | 8 | 8 | 8 | 8 | 8 | 8 | 8 | 7 | 7 | 8 | 8 | 8 | 8 | 8 | 8 | 7 | 8 | 8 | 7 |
| A Repeat | IGS *(psaC-ndhE)* | 10 | 11 | 4 | 8 | 6 | 6 | 6 | 3 | 8 | 5 | 10 | 12 | 13 | 6 | 5 | 6 | 9 | 5 | 2 | 3 | 3 |
| A Repeat | IGS *(ccsA-ndhD)* | 10 | 10 | 9 | 8 | 10 | 7 | 8 | 7 | 8 | 9 | 8 | 10 | 8 | 8 | 9 | 7 | 8 | 9 | 4 | 3 | 3 |
| A Repeat | IGS *(trnS-GGA-rps4)* | 10 | 16 | 9 | 10 | 10 | 8 | 9 | 10 | 12 | 4 | 10 | 10 | 12 | 10 | 8 | 7 | 8 | 4 | 7 | 8 | 8 |
| A Repeat | IGS *(petN-psbM)* | 10 | 8 | 4 | 6 | 4 | 3 | 4 | 8 | 8 | 4 | 8 | 10 | 8 | 4 | 4 | 4 | 4 | 4 | 4 | 3 | 3 |
| A Repeat | IGS *(rpoB-trnC-GCA)* | 10 | 9 | 4 | 5 | 5 | 5 | 5 | 9 | 10 | 4 | 8 | 9 | 11 | 5 | 5 | 6 | 4 | 4 | 2 | 2 | 2 |
| A Repeat | *atpF* intron | 10 | 12 | 10 | 10 | 6 | 9 | 7 | 8 | 9 | 6 | 12 | 9 | 9 | 6 | 6 | 10 | 9 | 6 | 4 | 6 | 13 |
| A Repeat | IGS *(trnV-GAC-rps7)* | 11 | 9 | 8 | 10 | 10 | 9 | 9 | 10 | 10 | 10 | 11 | 9 | 9 | 10 | 9 | 10 | 8 | 10 | 12 | 12 | 8 |
| A Repeat | IGS *(ndhF-rpl32)* | 11 | 10 | 7 | 9 | 8 | 7 | 9 | 8 | 10 | 10 | 7 | 11 | 12 | 8 | 9 | 7 | 8 | 10 | 6 | 6 | 6 |
| A Repeat | *clpP* exon 2 | 11 | 11 | 6 | 11 | 10 | 9 | 9 | 9 | 11 | 9 | 5 | 9 | 10 | 10 | 9 | 10 | 8 | 9 | 7 | 7 | 9 |
| A Repeat | *clpP* exon 2 | 11 | 16 | 7 | 12 | 10 | 10 | 10 | 9 | 10 | 11 | 15 | 11 | 10 | 9 | 10 | 12 | 10 | 11 | 13 | 9 | 11 |
| A Repeat | *clpP* exon 2 | 11 | 8 | 8 | 7 | 12 | 8 | 11 | 8 | 8 | 13 | 9 | 8 | 8 | 11 | 11 | 11 | 9 | 13 | 8 | 8 | 11 |
| A Repeat | IGS *(rpl20-rps12)* | 11 | 10 | 11 | 8 | 7 | 8 | 8 | 9 | 9 | 13 | 9 | 13 | 9 | 8 | 9 | 14 | 9 | 13 | 12 | 10 | 9 |
| A Repeat | IGS *(trnC-GCA-petN)* | 11 | 11 | 9 | 11 | 9 | 10 | 11 | 9 | 9 | 11 | 11 | 10 | 10 | 10 | 7 | 13 | 4 | 11 | 10 | 11 | 11 |
| A Repeat | IGS *(rpoB-trnC-GCA)* | 11 | 9 | 7 | 9 | 7 | 7 | 7 | 9 | 9 | 7 | 10 | 12 | 12 | 12 | 7 | 7 | 6 | 7 | 12 | 7 | 7 |
| A Repeat | IGS *(atpH-atpI)* | 11 | 5 | 7 | 9 | 10 | 9 | 9 | 8 | 7 | 7 | 10 | 8 | 10 | 6 | 9 | 12 | 7 | 7 | 5 | 5 | 5 |
| A Repeat | *trnG-UCC* intron | 11 | 9 | 9 | 9 | 9 | 9 | 9 | 9 | 10 | 9 | 10 | 9 | 9 | 8 | 9 | 7 | 9 | 9 | 9 | 7 | 7 |
| A Repeat | *rps16* intron | 11 | 12 | 9 | 10 | 7 | 10 | 3 | 6 | 7 | 11 | 7 | 11 | 12 | 7 | 10 | 8 | 4 | 11 | 11 | 7 | 13 |
| A Repeat | IGS *(ndhF-rpl32)* | 12 | 12 | 14 | 9 | 12 | 9 | 9 | 8 | 13 | 8 | 9 | 11 | 11 | 11 | 8 | 10 | 10 | 8 | 4 | 5 | 5 |
| A Repeat | IGS *(ndhF-rpl32)* | 12 | 11 | 8 | 11 | 5 | 9 | 13 | 11 | 7 | 13 | 10 | 15 | 9 | 5 | 10 | 6 | 9 | 13 | 6 | 6 | 6 |
| A Repeat | IGS *(rpoB-trnC-GCA)* | 12 | 10 | 10 | 10 | 8 | 10 | 10 | 7 | 9 | 12 | 8 | 13 | 10 | 10 | 10 | 5 | 8 | 12 | 12 | 6 | 8 |
| A Repeat | IGS *(rps16-trnQ-UUG)* | 12 | 7 | 0 | 5 | 3 | 7 | 6 | 4 | 7 | 7 | 7 | 7 | 7 | 3 | 7 | 8 | 1 | 7 | 1 | 1 | 1 |
| A Repeat | *clpP* exon 1 | 13 | 9 | 8 | 9 | 8 | 9 | 9 | 9 | 8 | 9 | 7 | 12 | 11 | 9 | 9 | 9 | 8 | 9 | 11 | 8 | 7 |
| A Repeat | IGS *(trnK-UUU-rps16)* | 13 | 6 | 9 | 7 | 4 | 8 | 8 | 11 | 7 | 8 | 7 | 0 | 7 | 4 | 10 | 10 | 9 | 8 | 12 | 6 | 10 |
| A Repeat | IGS *(accD-psaI)* | 14 | 10 | 8 | 8 | 8 | 14 | 10 | 8 | 9 | 9 | 9 | 10 | 8 | 12 | 8 | 11 | 7 | 9 | 7 | 9 | 8 |
| T Repeat | *ycf1* | 10 | 10 | 5 | 9 | 9 | 9 | 9 | 9 | 9 | 9 | 9 | 10 | 7 | 9 | 9 | 9 | 9 | 9 | 8 | 8 | 6 |
| T Repeat | *ycf1* | 10 | 12 | 6 | 10 | 10 | 10 | 10 | 10 | 10 | 10 | 12 | 10 | 10 | 10 | 10 | 10 | 10 | 10 | 6 | 6 | 6 |
| T Repeat | IGS *(rps15-ycf1)* | 10 | 11 | 6 | 9 | 7 | 7 | 8 | 4 | 10 | 8 | 9 | 9 | 8 | 7 | 8 | 7 | 7 | 8 | 5 | 5 | 5 |
| T Repeat | IGS *(ndhE_ndhG)* | 10 | 7 | 7 | 6 | 3 | 7 | 5 | 7 | 7 | 5 | 8 | 7 | 9 | 2 | 5 | 13 | 8 | 5 | 5 | 6 | 6 |
| T Repeat | IGS *(ndhF-rpl32)* | 10 | 12 | 10 | 9 | 0 | 9 | 10 | 11 | 9 | 8 | 9 | 12 | 4 | 0 | 8 | 8 | 6 | 8 | 7 | 7 | 7 |
| T Repeat | *ndhF* | 10 | 13 | 5 | 13 | 11 | 5 | 13 | 10 | 10 | 12 | 7 | 10 | 10 | 11 | 12 | 5 | 7 | 12 | 3 | 3 | 3 |
| T Repeat | *rpl16* intron | 10 | 5 | 11 | 9 | 8 | 12 | 9 | 11 | 12 | 11 | 10 | 5 | 9 | 8 | 9 | 6 | 10 | 11 | 7 | 9 | 7 |
| T Repeat | *clpP* exon 2 | 10 | 16 | 10 | 13 | 11 | 9 | 9 | 13 | 13 | 11 | 14 | 12 | 11 | 13 | 12 | 10 | 9 | 11 | 11 | 4 | 11 |
| T Repeat | IGS *(rps18-rpl20)* | 10 | 11 | 16 | 11 | 10 | 10 | 10 | 11 | 13 | 8 | 4 | 11 | 11 | 14 | 10 | 9 | 7 | 11 | 13 | 9 | 15 |
| T Repeat | IGS *(trnW-CCA-trnP-UGG)* | 10 | 13 | 9 | 9 | 9 | 8 | 9 | 9 | 10 | 11 | 10 | 11 | 10 | 4 | 10 | 8 | 10 | 11 | 7 | 8 | 7 |
| T Repeat | IGS *(ndhC-trnV-UAC)* | 10 | 9 | 10 | 13 | 11 | 9 | 10 | 9 | 7 | 11 | 10 | 9 | 10 | 9 | 9 | 10 | 10 | 11 | 6 | 8 | 6 |
| T Repeat | IGS *(trnG-GCC-trnfM-CAU)* | 10 | 11 | 2 | 1 | 10 | 1 | 10 | 10 | 11 | 8 | 8 | 10 | 1 | 6 | 6 | 6 | 5 | 11 | 1 | 0 | 4 |
| T Repeat | IGS *(trnE-UUC-trnT-GGU)* | 10 | 5 | 5 | 5 | 5 | 5 | 5 | 5 | 5 | 5 | 5 | 5 | 5 | 5 | 5 | 5 | 5 | 5 | 4 | 4 | 5 |
| T Repeat | IGS *(trnE-UUC-trnT-GGU)* | 10 | 10 | 8 | 9 | 9 | 10 | 9 | 9 | 9 | 13 | 10 | 10 | 12 | 10 | 15 | 10 | 9 | 13 | 2 | 2 | 2 |
| T Repeat | *rpoC1* intron | 10 | 10 | 9 | 10 | 9 | 8 | 11 | 11 | 10 | 11 | 8 | 8 | 9 | 14 | 10 | 8 | 7 | 11 | 10 | 7 | 10 |
| T Repeat | IGS *(trnS-GCU-trnG-UCC)* | 10 | 6 | 6 | 6 | 3 | 6 | 6 | 11 | 10 | 6 | 6 | 5 | 6 | 3 | 6 | 6 | 6 | 6 | 9 | 7 | 6 |
| T Repeat | *ycf1* | 11 | 11 | 14 | 11 | 11 | 12 | 11 | 11 | 11 | 14 | 11 | 11 | 11 | 11 | 14 | 11 | 14 | 14 | 11 | 11 | 11 |
| T Repeat | IGS *(rps15-ycf1)* | 11 | 9 | 7 | 7 | 6 | 9 | 7 | 7 | 8 | 7 | 10 | 11 | 7 | 7 | 6 | 8 | 8 | 7 | 10 | 7 | 7 |
| T Repeat | IGS *(rps12-trnV-GAC)* | 11 | 9 | 8 | 10 | 10 | 9 | 9 | 10 | 10 | 10 | 11 | 9 | 9 | 10 | 9 | 10 | 8 | 10 | 12 | 12 | 8 |
| T Repeat | IGS *(psbE-petL)* | 11 | 9 | 9 | 11 | 9 | 12 | 9 | 0 | 12 | 12 | 9 | 9 | 11 | 9 | 14 | 7 | 9 | 12 | 10 | 7 | 8 |
| T Repeat | IGS *(atpB-rbcL)* | 11 | 10 | 5 | 5 | 5 | 5 | 6 | 8 | 10 | 5 | 9 | 9 | 9 | 5 | 5 | 5 | 5 | 5 | 5 | 5 | 5 |
| T Repeat | *rpoC1* intron | 11 | 9 | 9 | 14 | 10 | 9 | 9 | 11 | 15 | 15 | 16 | 12 | 17 | 14 | 16 | 12 | 9 | 15 | 4 | 11 | 5 |
| T Repeat | *rpoC2* | 11 | 11 | 11 | 11 | 11 | 11 | 11 | 11 | 11 | 11 | 11 | 11 | 11 | 11 | 11 | 11 | 11 | 11 | 11 | 11 | 11 |
| T Repeat | *rpoC2* | 11 | 11 | 11 | 11 | 11 | 11 | 11 | 5 | 11 | 11 | 11 | 11 | 11 | 11 | 11 | 11 | 6 | 11 | 10 | 11 | 11 |
| T Repeat | *ycf1* | 12 | 12 | 15 | 12 | 15 | 15 | 15 | 12 | 12 | 15 | 12 | 12 | 12 | 15 | 15 | 12 | 9 | 15 | 11 | 15 | 13 |
| T Repeat | IGS *(rpl36-infA)* | 12 | 16 | 9 | 9 | 7 | 8 | 9 | 13 | 12 | 10 | 9 | 10 | 9 | 7 | 14 | 9 | 10 | 10 | 5 | 6 | 6 |
| T Repeat | IGS *(rps2-rpoC2)* | 12 | 12 | 11 | 6 | 10 | 11 | 10 | 5 | 10 | 11 | 4 | 11 | 10 | 9 | 10 | 12 | 8 | 11 | 11 | 10 | 9 |
| T Repeat | IGS *(trnT-GGU-psbD)* | 13 | 11 | 11 | 6 | 11 | 10 | 8 | 10 | 10 | 8 | 11 | 13 | 10 | 10 | 8 | 9 | 6 | 8 | 4 | 4 | 4 |
| T Repeat | *rpl16* intron | 14 | 11 | 4 | 12 | 11 | 3 | 10 | 12 | 10 | 11 | 10 | 9 | 11 | 15 | 12 | 3 | 4 | 11 | 5 | 5 | 5 |
| T Repeat | IGS *(matK-trnK-UUU)* | 14 | 12 | 11 | 7 | 10 | 9 | 11 | 11 | 14 | 11 | 10 | 12 | 9 | 10 | 10 | 11 | 8 | 11 | 10 | 8 | 10 |
| T Repeat | *trnL-UAA* intron | 15 | 6 | 6 | 6 | 6 | 6 | 6 | 13 | 14 | 6 | 6 | 7 | 6 | 6 | 6 | 6 | 6 | 6 | 5 | 6 | 6 |

Table S5: Gene loss and pseudogenization in *Allium* and related taxa of Allioideae.

| Name | Accession number | Evolutionary Line | Length | Loss | | Pseudogenization | | | | | | | | | | |
| --- | --- | --- | --- | --- | --- | --- | --- | --- | --- | --- | --- | --- | --- | --- | --- | --- |
|  |  |  |  | *infA* | *rpl22* | *rps2* | *matK* | *infA* | *rps16* | *rpl22* | *rpl23* | *accD* | *cemA* | *ycf2* | *ycf1* | *ycf15* |
| *Allium monanthum* | MH748538 | 1 | 154804 |  |  | o |  |  |  |  |  |  |  |  |  | o |
| *Allium paradoxum* | MH053150 | 1 | 145819 |  | o | o |  | o | o |  | o |  |  |  |  | o |
| *Allium ursinum* | MH157875 | 1 | 153252 |  |  | o |  |  |  |  |  |  |  |  |  | o |
| *Alium cernuum* | MT348440 | 1 | 153750 |  |  |  |  |  |  |  |  |  |  |  |  | o |
| *Allium monanthum* | MT348452 | 1 | 154306 | o | o | o |  | o |  |  |  |  |  |  |  | o |
| *Allium siculum* | MT348457 | 1 | 154225 |  |  | o | o | o |  |  |  |  |  |  |  | o |
| *Allium ovalifolium* | MH341457 | 2 | 155055 |  |  |  |  |  |  |  |  |  |  |  |  | o |
| *Allium pratii* | MG739457 | 2 | 154482 |  |  | o |  |  |  |  |  |  |  |  |  | o |
| *Allium victorialis* | MF687749 | 2 | 154074 |  |  |  |  | o |  |  |  |  |  |  |  | o |
| *Allium spicatum* | MT348453 | 2 | 152822 | o |  | o | o |  | o |  |  |  |  |  |  | o |
| *Allium karataviense* | MT348442 | 2 | 151808 | o |  | o | o |  |  |  |  |  |  |  |  | o |
| *Allium nerinifolium* | MT348443 | 2 | 154129 |  |  |  |  |  | o |  |  |  |  | o |  | o |
| *Allium ochotense* | MT348451 | 2 | 154048 |  |  |  |  | o |  |  |  |  |  |  |  | o |
| *Allium tricoccum* | MT348456 | 2 | 153565 |  |  |  |  | o |  |  |  |  |  |  |  | o |
| *Allium cepa* | KM088013 | 3 | 153529 |  |  | o |  |  |  |  |  |  |  |  |  | o |
| *Allium altaicum* | MH159130 | 3 | 153129 |  |  | o |  |  |  |  |  |  |  |  |  | o |
| *Allium ampeloprasum* | MK820026 | 3 | 152732 | o |  | o |  |  | o |  |  |  |  |  |  | o |
| *Allium caeruleum* | MN648630 | 3 | 152967 | o |  | o |  |  |  |  |  |  |  |  |  | o |
| *Allium cyathophorum* | MH341454 | 3 | 153568 |  |  | o |  |  | o |  |  |  |  | o |  | o |
| *Allium chinense* | MK096442 | 3 | 152525 |  |  |  |  |  |  | o |  |  |  |  |  | o |
| *Allium chrysanthum* | MH992108 | 3 | 153621 |  |  | o |  |  |  |  |  |  |  |  |  | o |
| *Allium delicatulum* | MN648631 | 3 | 152984 |  |  | o |  |  |  |  |  |  |  |  |  | o |
| *Allium fistulosum* | MH926357 | 3 | 153164 |  |  | o |  |  |  |  |  |  |  |  |  | o |
| *Allium herderianum* | MH992110 | 3 | 153605 |  |  | o |  |  |  |  |  |  |  |  |  | o |
| *Allium macrostemon* | MK751472 | 3 | 153158 | o |  | o |  |  |  |  |  |  |  |  |  | o |
| *Allium mairei* | MK820615 | 3 | 152913 |  |  | o |  |  |  |  |  |  |  |  |  | o |
| *Allium maowenense* | MH992111 | 3 | 153608 |  |  | o |  |  |  |  |  |  |  |  |  | o |
| *Allium obliquum* | MG670111 | 3 | 152387 |  |  | o |  |  | o |  |  |  |  |  |  | o |
| *Allium oschaninii* | MK411816 | 3 | 153580 |  |  | o |  |  |  |  |  |  |  |  |  | o |
| *Allium pallasii* | MN648632 | 3 | 151672 |  |  | o |  |  | o |  |  |  |  |  |  | o |
| *Allium praemixtum* | MK411817 | 3 | 153226 |  |  | o |  |  |  |  |  |  |  |  |  | o |
| *Allium przewalskianum* | MN519210 | 3 | 153509 |  |  | o |  |  |  |  |  |  |  |  |  | o |
| *Allium pskemense* | MK411815 | 3 | 153788 |  |  |  |  |  |  |  |  |  |  |  |  | o |
| *Allium ramosum* | MH159131 | 3 | 154034 | o |  | o |  |  |  |  |  |  |  |  |  | o |
| *Allium rude* | MH992112 | 3 | 153697 |  |  | o |  |  |  |  |  |  |  |  |  | o |
| *Allium sativum* | KY363332 | 3 | 153131 |  |  | o |  |  |  |  |  |  |  |  |  | o |
| *Allium spicatum* | MK931246 | 3 | 153187 |  |  | o |  |  |  |  |  |  |  |  |  | o |
| *Allium tanguticum* | MN648635 | 3 | 153024 |  |  | o |  |  |  |  |  |  |  |  |  | o |
| *Allium tuberosum* | MK335929 | 3 | 154056 | o |  | o |  |  |  |  |  |  |  |  |  | o |
| *Allium xichuanense* | MH992113 | 3 | 153673 |  |  | o |  |  |  |  |  |  |  |  |  | o |
| *Allium nigrum* | MT348458 | 3 | 152241 |  |  | o |  |  |  |  |  | o |  |  |  | o |
| *Allium koreanum* | MT348449 | 3 | 153162 |  |  | o |  |  |  |  |  |  |  |  |  | o |
| *Allium schoenprasum* | MT348444 | 3 | 152852 |  |  |  |  |  | o |  |  |  |  |  |  | o |
| *Allium senescens* | MT348450 | 3 | 153542 |  |  | o |  |  |  |  |  |  |  |  |  | o |
| *Allium cyathophorum* | MT348441 | 3 | 153503 |  |  | o |  |  |  |  |  |  |  |  |  | o |
| *Gillesia graminea* | MT348447 | - | 154067 |  |  |  |  |  |  |  |  |  |  |  | o | o |
| *Nothoscordum bonariense* | MT348455 | - | 156559 |  |  |  |  |  |  |  |  |  |  |  |  | o |
| *Tulbaghia violaceae* | MT323239 | - | 156449 |  |  |  |  | o | o |  | o |  | o |  |  | o |
| *Cyrtanthus mackenii* | MT348446 | - | 159998 |  |  |  |  |  |  |  |  |  |  |  |  | o |
| *Calostemma purpureum* | MT348445 | - | 159500 |  |  |  |  |  |  |  |  |  |  |  |  | o |
| *Lycoris radiata* | MT348454 | - | 158355 |  |  |  |  |  |  |  |  |  |  |  |  | o |
| *Zephyranthes mesochloa* | MT323238 | - | 158768 |  |  |  |  |  |  |  |  |  |  |  |  | o |
| *Crinum asiaticum var. pedunculatum* | MT348448 | - | 158683 |  |  |  |  |  |  |  |  |  |  |  |  | o |
| *Agapanthus coddii* | MT348439 | - | 156699 |  |  |  |  |  |  |  |  |  |  |  |  | o |
| The circles indicate the presence of gene loss and pseudogenization | | | | | | | | | | | | | |  |  |  |

Table S6: Comparison of divergence times of Allioideae and its tribes in different studies.

| Node | | Our study | Chen et al. (2013) | Sassone & Giussani (2018) | Costa et al. (2020) | Xie et al. (2020) | Escobar et al. (2020) |  |
| --- | --- | --- | --- | --- | --- | --- | --- | --- |
| Allioideae | | 40.1 (28.5-55.3) Mya | 37 (27.8-44.5) Mya | - | 63.5 (53.7-67.5) Mya | 41.932 (34.5-47.6) Mya | 64.187 Mya |  |
| Allieae | | 21.3 (14.4-28.8) Mya | - | 33,97 Mya | 52.2 (44.4-58.1) Mya | - | 57.421 Mya |  |
| Gilliesieae + Leucocoryneae + Tulbaghieae | | 25.3 (11.5-39.1) Mya | - | 37,51 Mya | 54.1 (37.11-65.1) Mya | - | - |  |
| Gilliesieae + Leucocoryneae | | 16.5 (5.0-28.5) Mya | - | 31,77 Mya | 45 (32.2-61.2) Mya | - | 50.01 Mya |  |
| Data | | 74 coding sequences of chloroplast genomes | *matK, ndhF, rbcL,* and *atpB* of chloroplast genomes | Nuclear ribosomal ITS and plastid (*ndhF* and matK) sequences | ITS of nuclear; *matK, ndhF*, and *rbcL* of chloroplast genomes | 48 shared single copy genes | Nuclear ribosomal ITS; plastid (*rbcL* + *trnL-F*) |  |
|  | The dash “-“ means no data | | | | | | | |

| Table S7: List of species used for comparative genomics. | | | | | | | | |
| --- | --- | --- | --- | --- | --- | --- | --- | --- |
| Family | Subfamily | Tribe | Subgenus  (sensu Friesen et al., 2006) | species | Total Read | Number of Sequence (%) | Coverage | Voucher (Herbarium) |
| Amaryllidaceae | Allioideae | Allieae | *Aguinum* | *Allium victorialis* | - | - | - | MF687749 |
|  |  |  | *Aguinum* | *A. tricoccum* | 11497496 | 49470 (0.43%) | 96.7x | Namgung 20120529 (WIS) |
|  |  |  | *Aguinum* | *A. ochotense* | 22588638 | 31544 (0.14%) | 20.5x | Kim 04-142 (GCU) |
|  |  |  | *Allium* | *A. senescens* | 10832788 | 88875 (0.82%) | 173.4x | Namgung 20170811 (GCU) |
|  |  |  | Amerallium | *A. ursinum* | - | - | - |  |
|  |  |  | *Amerallium* | *A. cernuum* | 10709302 | 53565 (0.5%) | 88.5x | Namgung 20170811 (WIS) |
|  |  |  | *Caloscordum* | *A. neriniflorum* | 9989094 | 210989 (2.11%) | 410.5x | Brownless 19841350 (RBGE) |
|  |  |  | *Cepa* | *A. cepa* | - | - | - | KM088013 |
|  |  |  | *Cepa* | *A. schoenoprasum* | 3668798 | 62449 (1.7%) | 123x | Choi 20150714 (CWU) |
|  |  |  | *Cyathophora* | *A. cyathophorum* | 10429892 | 104697 (1%) | 149.5x | Brownless 20001464 (RBGE) |
|  |  |  | *Cyathophora* | *A. spicatum* | 7081036 | 48990 (0.69%) | 85.6x | Choi 20160513 (CWU) |
|  |  |  | *Melanocrommyum* | *A. karataviense* | 13386084 | 156294 (1.16%) | 308.8x | Choi 11052015-8 (CWU) |
|  |  |  | *Melanocrommyum* | *A.nigrum* | 10734306 | 76482 (0.71%) | 15.8x | Brownless 19570130 (RBGE) |
|  |  |  | Microscordum | *A. monanthum* | 9898464 | 142210 (1.43%) | 197x | Namgung 309921 (NIBR) |
|  |  |  | Nectaroscordum | *A. siculum* | 11405230 | 165603 (1.45%) | 322x | Chase 152 (Kew) |
|  |  |  | *Polyprason* | *A. obliquum* | - | - | - | NC037199 |
|  |  |  | *Reticulatobulbosa* | *A. koreanum* | 4011298 | 50577 (1.26%) | 99.1x | Choi 2015622 (CWU) |
|  |  |  | *Rhizirideum* | *A. sativum* | - | - | - | NC031829 |
|  |  | Leucocoryneae | - | *Nothoscordum bonariense* | 9933020 | 153916 (1.54%) | 295.1x | Brownless 19811006 (RBGE) |
|  |  | Tulbaghinea | - | *Tulbagia violacea* | 9368352 | 18366 (0.19%) | 27.9x | Namgung 20181023 (SCHG) |
|  |  | Gilliesieae | - | *Gillesia graminea* | 13281774 | 273901 (2.06%) | 533.2x | Chase 450 (Kew) |
|  | Agapanthoideae | - | - | *Agapanthus coddii* | 39000698 | 30928 (0.79%) | 65.8x | Chase 20081397 (Kew) |
|  | Amaryllioideae | - | - | *Calostemma purpureum* | 12103602 | 110932 (0.91%) | 211x | Namgung 20180430 (GCU) |
|  |  | - | - | *Crinum asiaticum* var *pendunculatum* | 9674986 | 48066(0.50%) | 91x | Park 140806-1 (GCU) |
|  |  | - | - | *Cyrtanthus mackenii* | 3923266 | 80474 (2.05%) | 133.4x | Namgung 20171110 (KNA) |
|  |  | - | - | *Lycoris radiata* | 11366508 | 102700 (0.9%) | 194.3x | Hasz 20421 (DAV) |
|  |  | - | - | *Zephyranthes mesochloa* | 11779992 | 189362 (1.06%) | 358.1x | Brownless 19871199 (RBGE) |
| Asparagaceae | Asparagoideae | - | - | *Asparagus officinalis* | - | - | - | NC034777 |
|  | Agavoideae | - | - | *Hosta yingeri* | - | - | - | NC039976 |
|  |  | - | - | *Yucca filamentosa* | - | - | - | KX931467 |
| Xanthorrhoreaceae | Xanthorrhoreoideae | - | - | *Xanthorrhorea preissii* | - | - | - | NC_035996 |
| Iridaceae | Irioideae | - | - | *Iris sanguinea* | - | - | - | KT626943 |
| The dash "-" means no information | |  |  |  |  |  |  |  |
